# Hydrocarbon constrained peptides – understanding preorganisation and binding affinity†
†Electronic supplementary information (ESI) available. CCDC 1057141. For ESI and crystallographic data in CIF or other electronic format see DOI: 10.1039/c5sc04048e



**DOI:** 10.1039/c5sc04048e

**Published:** 2016-02-29

**Authors:** Jennifer A. Miles, David J. Yeo, Philip Rowell, Silvia Rodriguez-Marin, Christopher M. Pask, Stuart L. Warriner, Thomas A. Edwards, Andrew J. Wilson

**Affiliations:** a School of Chemistry , University of Leeds , Woodhouse Lane , Leeds LS2 9JT , UK . Email: A.J.Wilson@leeds.ac.uk; b Astbury Centre for Structural Molecular Biology , University of Leeds , Woodhouse Lane , Leeds LS2 9JT , UK . Email: T.A.Edwards@leeds.ac.uk; c School of Molecular and Cellular Biology , University of Leeds , Woodhouse Lane , Leeds LS2 9JT , UK

## Abstract

The development of constrained peptides represents an emerging strategy to generate peptide based probes and hits for drug-discovery that address challenging protein–protein interactions (PPIs). In this manuscript we report on the use of a novel α-alkenylglycine derived amino acid to synthesise hydrocarbon constrained BH3-family sequences (BIM and BID). Our biophysical and structural analyses illustrate that whilst the introduction of the constraint increases the population of the bioactive α-helical conformation of the peptide in solution, it does not enhance the inhibitory potency against pro-apoptotic Bcl-x_L_ and Mcl-1 PPIs. SPR analyses indicate binding occurs *via* an induced fit mechanism whilst X-ray analyses illustrate none of the key interactions between the helix and protein are disturbed. The behaviour derives from enthalpy–entropy compensation which may be considered in terms of the ground state energies of the unbound constrained and unconstrained peptides; this has implications for the design of preorganised peptides to target protein–protein interactions.

## Abstract 2

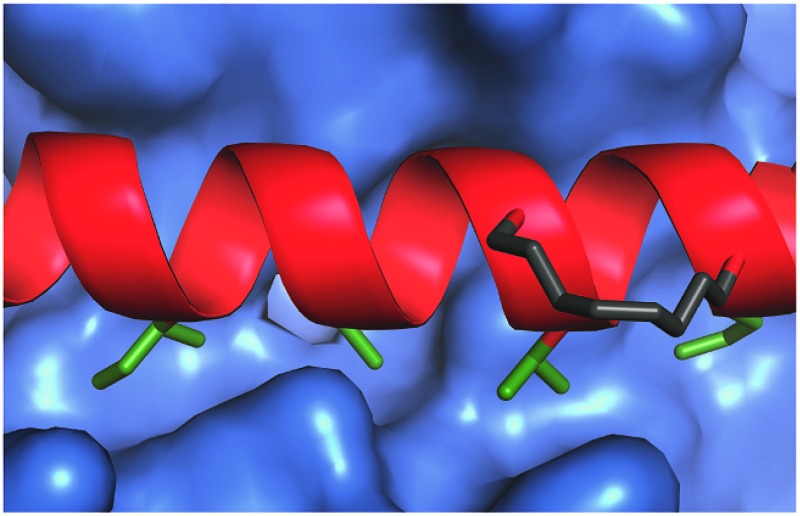
Biophysical studies on hydrocarbon constrained peptides reveal induced fit binding and enthalpy–entropy compensation on target protein recognition.

## Introduction

Protein–protein interactions (PPIs) mediate virtually all biological processes and as such there is a demand for methods that can modulate these interactions in a selective, dose-dependent and temporal manner. PPIs are considered challenging targets for molecular intervention,^1^ as they typically involve interaction of large, comparatively featureless protein surfaces. Inhibition of PPIs mediated by α-helices represents an area of chemical biology that has become tractable in terms of ligand development, with a variety of small molecule, constrained peptides and helix mimetics under development.^2^ Constraining peptides in a helical conformation has been reported to confer benefits that include enhanced protease resistance, stability in cells, increased cellular uptake^3^ and enhanced biophysical properties in comparison to wild-type peptide sequences.^4,5^ More specifically, constrained peptides are anticipated to bind their targets with higher potency due to a reduced entropic cost of adopting a bioactive α-helical conformation. Multiple methods have been used to constrain peptides,^6^ that make use of helix favouring amino acids,^7,8^ helix nucleating motifs,^9^ disulfides,^10^ lactam bridges,^11,12^ hydrogen bonding surrogates,^13,14^ hydrocarbon linkages (colloquially termed “staples”)^4,5,15–20^ and other modifications;^21–27^ proline is particularly noteworthy as a helix breaker^28,29^ but may also act to cap a helix.^30^ Hydrocarbon constraints are incorporated by replacing native amino acids with an α-α-disubstituted alkenyl amino acids followed by ruthenium catalysed olefin metathesis to create the irreversible constraint.^31–33^ By carefully choosing the residues to replace, one should be able to create the constraint whilst maintaining the essential residues required for interaction with the target protein.

**Fig. 1:**
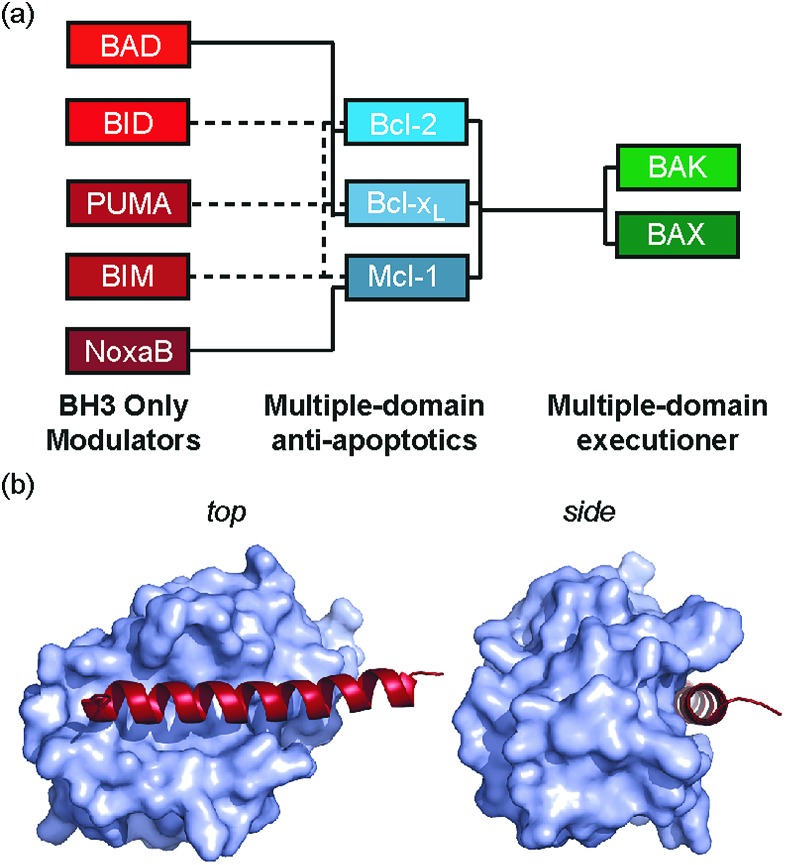
Bcl-2 family PPIs (a) schematic depicting selectivity preferences for interaction of the modulators and pro-apoptotic family members with the anti-apoptotic family members (dashed lines denote promiscuous recognition, full lines denote selective interactions). (b) Crystal structure (PDB ID: 3FDL) of Bcl-x_L_/BIM interaction (Bcl-x_L_ as a surface and BIM BH3 peptide shown in red).

We recently introduced the monosubstituted amino alkenyl amino acid as an effective reagent for creation of a hydrocarbon constraint.^34^ This amino acid should couple more effectively in conventional Fmoc mediated peptide synthesis and the resultant constraint may be advantageous over the disubstituted amino acid in terms of removing steric clashes with the target.^35^ In this work we investigate the use of a monosubstituted amino acid in the creation of a hydrocarbon constraint using Bcl-2/BH3 family PPIs as a model (Fig. 1a and b). This family of proteins play a pivotal role in the regulation of apoptosis,^36^ play a major role in cancer development/progression^37^ and represent key targets for anticancer drug-discovery^38^ The regulation of apoptosis exploits varying specificity and selectivity of pairwise interactions within the Bcl-2 family interactome (Fig. 1a).^39,40^ The canonical structural motif involves a BH3 domain from one Bcl-2 family member adopting a helical conformation and binding to a BH3 binding cleft of another partner (Fig. 1b). In our original study, we observed an increase in peptide helicity and enhanced enzymatic stability with the use of the mono-substituted amino acid for one member of the Bcl-2 effector sub family (BID). In our hands and in contrast to prior proof-of-concept studies,^15,41^ the potency of inhibition for Bcl-x_L_/BAK was comparable to that of the wild type (WT), and variant peptides bearing aminoisobutyric acid or the disubstituted amino acid at the stapling positions.^34^ The purpose of this current study was to understand whether this behaviour was unique to BID and to establish a more detailed explanation for the seemingly paradoxical observation that a net increase in the population of the bioactive conformation failed to yield an increase in the binding affinity to the target protein.

## Results

### Synthesis

In our initial investigation we introduced a hydrocarbon constraint into the BID-BH3 peptide to compare the use of a monosubstituted amino acid with the more widely exploited disubstituted amino acid.^34^ We synthesised (*S*)-pentenylglycine using the method developed by Belokon and co-workers;^42^ the stereochemistry of the amino acid was confirmed by single crystal diffraction studies on the precursor nickel complex (see ESI†). For a full comparison, we also synthesized the disubstituted amino acid and incorporated this, the monosubstituted amino acid and aminoisobutyric acid (Aib) at appropriate positions in the BID sequence (replacing ^92^Gln and ^96^Ser). In the current study a series of additional peptides were either sourced commercially or synthesized in house (Table 1) including Bcl-2 family WT peptides, peptides labelled with a fluorescent group (fluorescein or BODIPY) to facilitate direct binding measurements and a series of constrained BIM peptides. We selected BIM BH3, one of the key BH3 members, as it has a high affinity for all of the anti-apoptotic proteins (Fig. 1a).^39,40^ Peptide “staples” were introduced at positions previously identified as optimal.^15,41,43^ The peptides were prepared using standard Fmoc solid phase peptide synthesis protocols using low loading Rink amide MBHA resin to afford C-terminally amidated peptides.^34^ Alkenyl amino acid containing peptides were subjected to olefin metathesis to generate constrained peptides. In order to prevent any complications arising from interaction of the Grubbs' catalyst with the FITC label, the metathesis was performed before the final Fmoc deprotection and coupling of FITC. Constrained BIM peptides were synthesised by replacing ^94^Arg and ^98^Glu with non-natural amino acids.^44^ BIM-DM (disubstituted metathesized) and BIM-MM (monosubstituted metathesized) peptides were synthesized on a 0.075 or a 0.1 mmol scale respectively; the first 8 residues were built using an automated peptide synthesiser and, subsequently, unnatural amino acids and residues in between were coupled manually with the remaining 8 residues coupled using automated methods. After on-resin Grubbs' metathesis, the BIM-MM and BIM-DM peptides were obtained in high purity with no observed deletions and purified using semi-preparative HPLC.

**Table 1:** Peptide sequences used in this work. (WT – wild-type, MM – monosubstituted metathesised, DM – disubstituted metathesised, AIB – aminoisobutyric acid substituted, Ahx – amino hexanoic acid)

Peptide	Sequence
BAK WT	Ac-Ahx-G^72^QVGRQLAIIGDDINR^87^-NH_2_
BID WT	Ac-E^80^DIIRNIARHLAQVGDSN_L_DRSIW^102^-NH_2_
BAD WT	Ac-L^104^WAAQRYGRELRRMSDEFEGSFDKL^128^-NH_2_
BIM-WT	Ac-I^56^WIAQELRRIGDEFNAYYARR^56^-NH_2_
NOXA-B WT	Ac-P^68^ADLKDECAQLRRIGDKVNL^87^-NH_2_
BID-MM	Ac-E^80^DIIRNIARHLAXVGDXN_L_DRSIW^102^-NH_2_
BID-DM	Ac-E^80^DIIRNIARHLAZVGDZN_L_DRSIW^102^-NH_2_
BID-AIB	Ac-E^80^DIIRNIARHLA(Aib)VGD(Aib)N_L_DRSIW^102^-NH_2_
BIM-MM	Ac-I^56^WIAQELRXIGDXFNAYYARR^76^-NH_2_
BIM-DM	Ac-I^56^WIAQELRZIGDZFNAYYARR^76^-NH_2_
BODIPY-BAK	BODIPY-Ahx-G^72^QVGRQLAIIGDDINR^87^-NH_2_
FITC-BID-WT	FITC-Ahx-E^80^DIIRNIARHLAQVGDSN_L_DRSIW^102^-NH_2_
FITC-BID-MM	FITC-βAla-E^80^DIIRNIARHLAXVGDXN_L_DRSIW^102^-NH_2_
FITC-BID-DM	FITC-βAla-E^80^DIIRNIARHLAZVGDZN_L_DRSIW^102^-NH_2_
FITC-BID-AIB	FITC-βAla-E^80^DIIRNIARHLA(Aib)VGD(Aib)N_L_DRSIW^102^-NH_2_
FITC-NOXA-B WT	FITC-Ahx-P^68^ADLKDECAQLRRIGDKVNL^87^-NH_2_
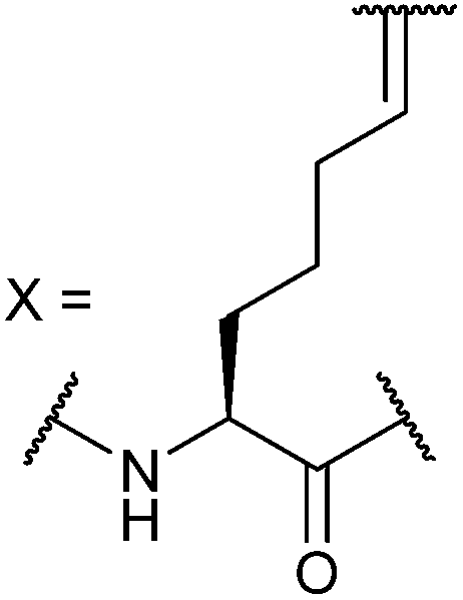	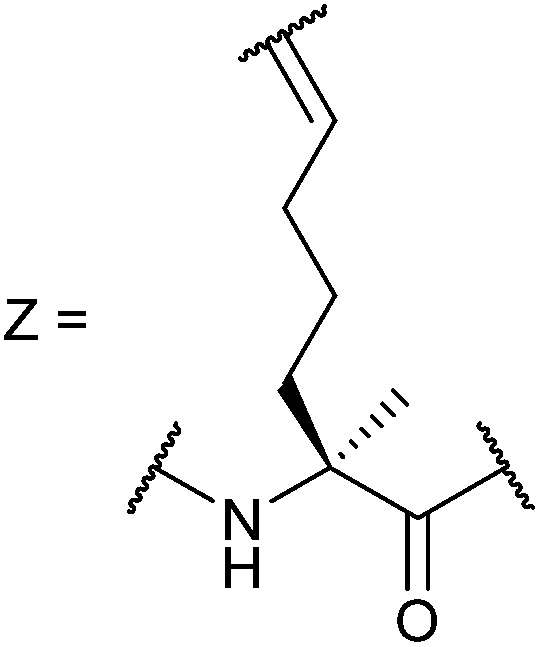

### Peptide conformation

**Fig. 2:**
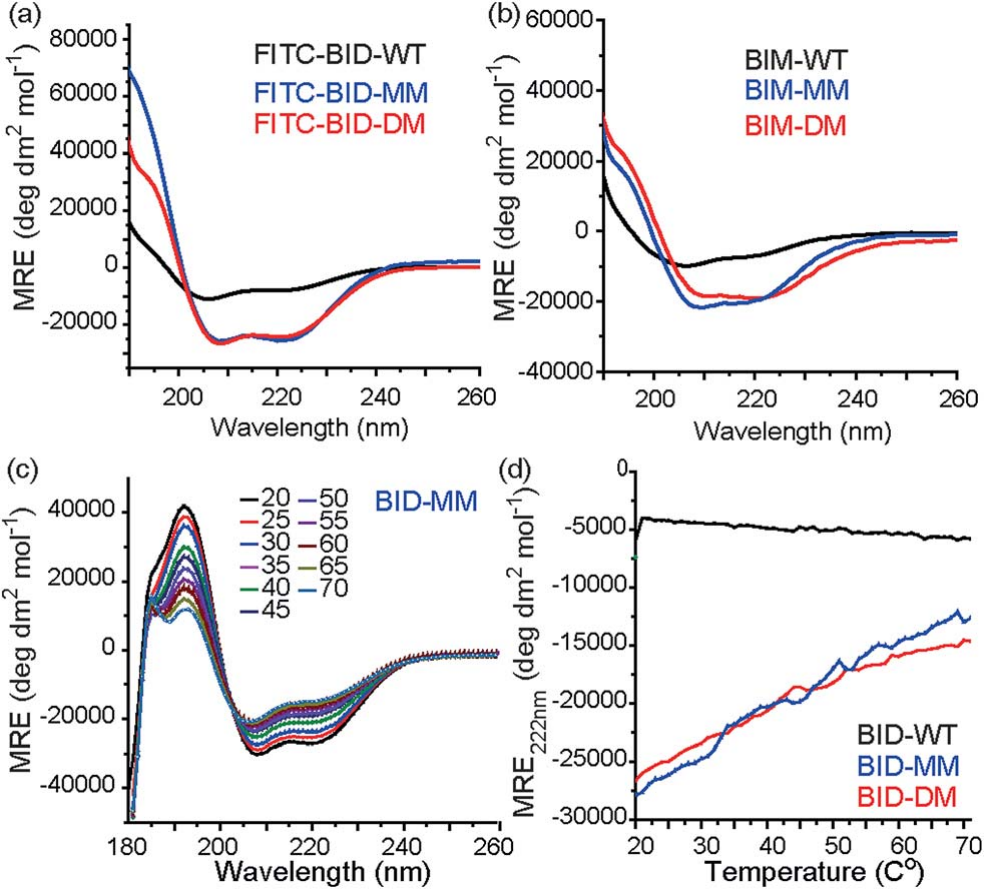
Conformational analysis of BH3 peptides by circular dichroism (CD). (a) Fluorescein labelled BID peptides (b) BIM peptides (c) thermal unfolding CD spectra for BID-MM. (d) Changes in MRE value at 222 nm for BID peptides with temperature.

Consistent with our preliminary report, incorporation of a constraint in the BID or BIM peptides induced helicity, independent of the use of a mono or disubstituted amino acid and was not affected by the presence of FITC. FITC labelled peptides retained the enhanced helicity in circular dichroism (CD) spectra observed for their unlabelled counterparts (Fig. 2a), with 74% (FITC-BID-MM) and 70% (FITC-BID-DM) helicity in comparison to the FITC-BID-WT, which had 23% helicity. The spectra were found to be concentration independent (see ESI†). Making an assumption that the difference in helicity is proportional to the difference in stability between constrained and WT allows ΔΔ*G* ∼ 2.9 kJ mol^–1^ to be estimated for introduction of the hydrocarbon constraint. The constrained BIM peptides also showed an increase in helicity as determined by CD, with 57% of BIM-DM and 59% helicity within BIM-MM in comparison to the BIM-WT of 20% (Fig. 2b). Again making assumptions as for BID allowed ΔΔ*G* ∼ 2.6 kJ mol^–1^ to be estimated for introduction of the constraint.

Thermal unfolding experiments were carried out to understand how well the constraint maintains the helical conformation upon denaturation. The mean residue ellipticity was compared against the unfolded wild type as a function of temperature to evaluate the extent that it resists unfolding. There were minor changes in signal at 222 nm of both BID-MM and BID-DM, whereas the wild-type BID peptide was mostly unfolded at the start of the experiment (Fig. 2c, d). Due to the presence of the hydrocarbon linker, constrained peptides could not be completely unfolded into a random coil conformation; the thermal unfolding reduced the helical content of the constrained BID peptides to 37–40% (Fig. 2d). Notably, the transition does not progress through a sigmoidal curve characteristic of two state co-operative unfolding; this gradual transition is expected for a short helix with low enthalpy of folding.^45^

### Binding of constrained peptides to Bcl-2 family members

**Fig. 3:**
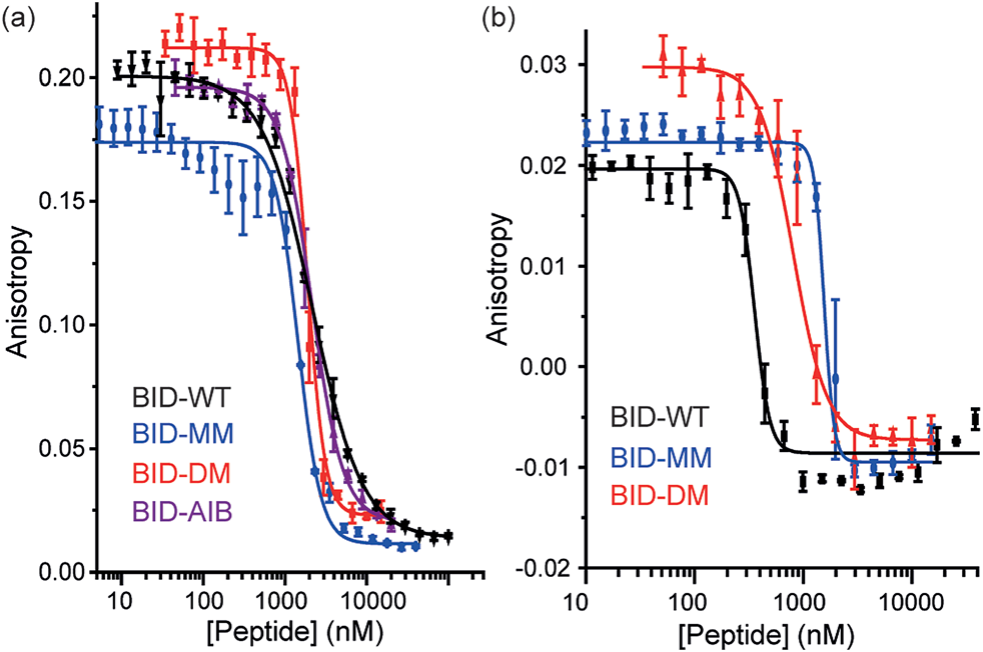
BID peptide/protein titrations (a) fluorescence anisotropy competition assay for inhibition of Bcl-x_L_/BAK interaction, with Bcl-x_L_ at 131 nM and BODIPY-BAK at 43 nM (b) fluorescence anisotropy competition assay for inhibition of Mcl-1/NOXA-B interaction, with Mcl-1 at 150 nM and FITC-NOXA-B at 50 nM (40 mM sodium phosphate, 200 mM sodium chloride, 0.02 mg ml^–1^ bovine serum albumin, pH 7.50).

To profile the binding behaviour of the constrained peptides we tested them in both competition displacement assays and direct binding assays. We selected Bcl-x_L_ and Mcl-1 as targets because they have differential binding properties towards BH3 only sequences.^39,40^ Of particular interest was the potential for the introduction of a constraint to have differential effects on protein specificity/selectivity. Initially, we confirmed prior results by testing the binding of labelled WT BH3 peptides (BAK, BID and NOXA-B) to Bcl-x_L_ and Mcl-1 using fluorescence anisotropy (FA) and through FA competition assays in which unlabelled peptides are used to displace labelled BAK or NOXA-B from the Bcl-x_L_ or Mcl-1 respectively (see ESI†). We next tested the constrained peptides in inhibition assays (Fig. 3a and b and Table 2). For BID, no effect on inhibitory potency against Bcl-x_L_/BODIPY-BAK in comparison to the BID-WT was observed (BID-WT 1.44 μM, BID-MM 0.62 μM, BID-DM 1.14 μM and the BID-Aib 1.14 μM). We then tested BID-MM and BID-DM against Mcl-1/FITC-NOXA-B in a FA based competition assay. In competition against this different Bcl-2/BH3 pairing, BID-MM was less potent, with an IC_50_ of 1.6 μM in comparison to 0.39 μM with BID-WT, whilst BID-DM was slightly better than BID-MM, with an IC_50_ of 0.8 μM.

**Table 2:** Fluorescence anisotropy competition, fluorescence anisotropy direct binding experiments for BID and BIM peptides

BH3 peptide	Bcl-x_L_/BODIPY-BAK IC_50_	Mcl-1/FITC-NOXA-B IC_50_
BID-WT	1.44 ± 0.05 μM	0.39 ± 0.08 μM
BID-MM	0.62 ± 0.02 μM	1.6 ± 0.12 μM^*a*^
BID-DM	1.14 ± 0.04 μM	0.8 ± 0.07 μM ^*a*^
BID-AIB	1.14 ± 0.05 μM	N/D
BIM-WT	320 ± 70 nM	50 ± 2 nM
BIM-MM	11.9 ± 3.8 μM^*a*^	1.8 ± 0.25 μM^*a*^
BIM-DM	4 ± 0.62 μM^*a*^ ^,^ ^*b*^	890 ± 150 nM^*a*^ ^,^ ^*b*^

	Bcl-x_L_ EC_50_	Mcl-1 EC_50_
FITC-BID-WT	79 ± 6 nM	98 ± 8.9 nM
FITC-BID-MM	186 ± 24 nM	153 ± 12 nM
FITC-BID-DM	159 ± 13 nM	129 ± 3 nM
FITC-BID-Aib	57 ± 16 nM	84 ± 13 nM

^*a*^Two tailed *p* value with WT < 0.01.

^*b*^Two tailed *p* value with MM < 0.02.

**Fig. 4:**
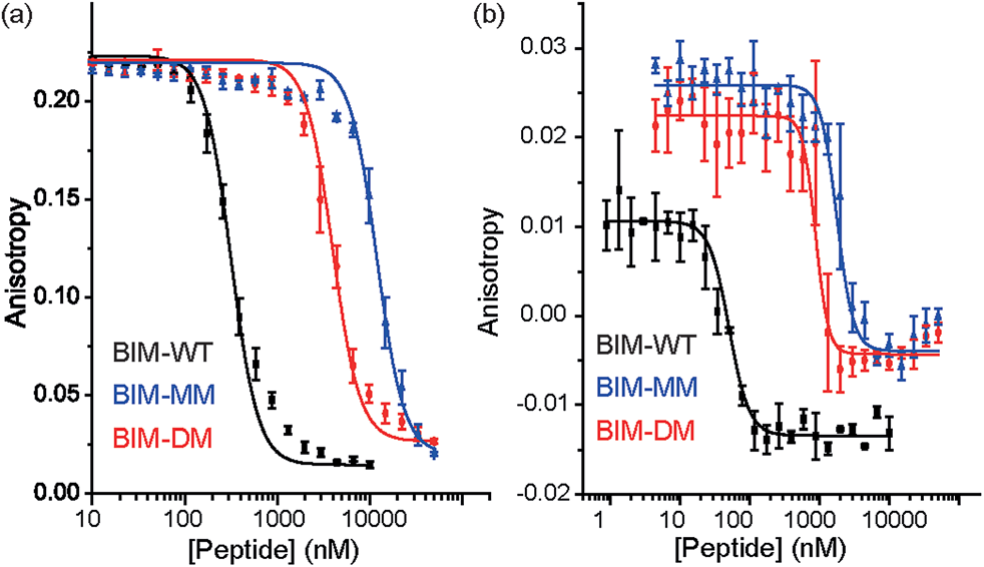
BIM peptide/protein titrations (a) fluorescence anisotropy competition assay for inhibition of Bcl-x_L_/BAK interaction, with Bcl-x_L_ at 131 nM and BODIPY-BAK at 43 nM (b) fluorescence anisotropy competition assay for inhibition of Mcl-1/NOXA-B interaction, with Mcl-1 at 150 nM and FITC-NOXA-B at 50 nM (40 mM sodium phosphate, 200 mM sodium chloride, 0.02 mg ml^–1^ bovine serum albumin, pH 7.50).

The constrained BIM peptides were also tested in competition assays against Bcl-x_L_/BODIPY-BAK and Mcl-1/FITC-NOXA-B interactions. In the case of Bcl-x_L_/BODIPY-BAK, the constrained BIM variants were less potent, IC_50_ = 320 nM for BIM-WT, IC_50_ = 11.9 μM for BIM-MM and IC_50_ = 4 μM BIM-DM (Fig. 4a). This result was surprising, given that the literature reports a 3-fold improvement in potency for stapling the BIM peptide.^15,41^ The constrained BIM peptides were also significantly worse at inhibiting the Mcl-1/FITC-NOXA-B interaction, with IC_50_ values of 1.8 μM for BIM-MM and IC_50_ of 890 nM for BIM-DM compared to an IC_50_ of 50 nM for BIM-WT (Fig. 4b).

**Fig. 5:**
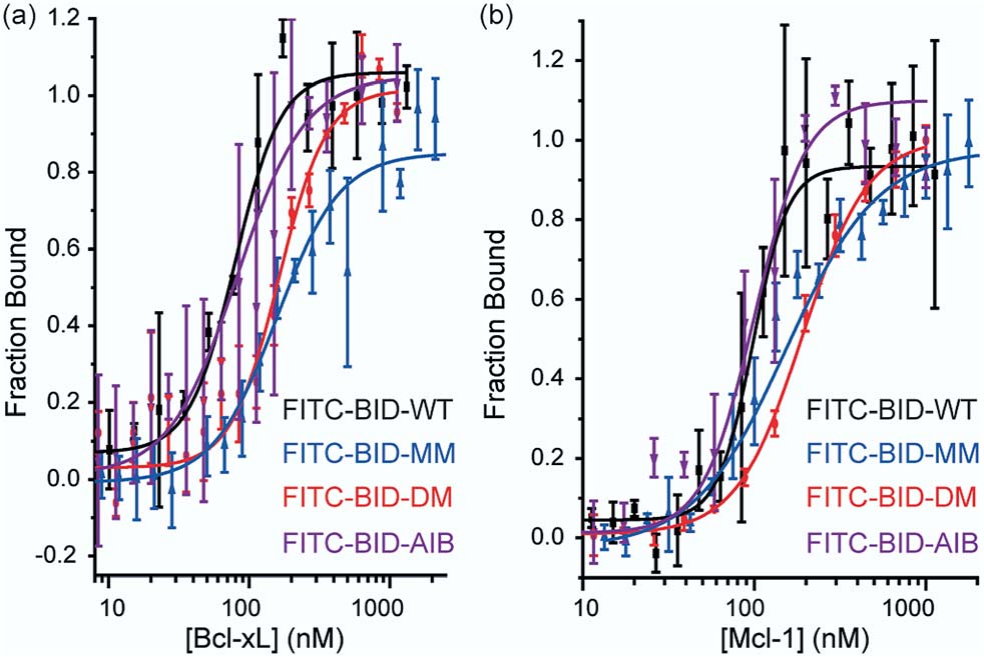
BID peptide/protein titrations (a) fluorescence anisotropy direct binding assay for interaction of BH3 peptides with Bcl-x_L_ (b) fluorescence anisotropy direct binding assay for interaction of BH3 peptides with Mcl-1 (50 mM tris, 140 mM sodium chloride, pH 7.50).

Direct titration experiments were also performed for FITC labelled BID peptides (Fig. 5). Fluorescence anisotropy was used to measure direct binding with Bcl-x_L_ titrated into each of the FITC-labelled-modified-BID peptides. The data did not fit well to a 1 : 1 binding isotherm in these conditions, indicating the presence of non-specific binding phenomena (possibly due to the fluorophore) and hence was fitted using a logistic model. FITC-BID-WT had an EC_50_ of 79 nM, with FITC-BID-MM and FITC-BID-DM observed to bind with slightly reduced affinity; EC_50_ values of 186 nM and 159 nM. FITC-BID-Aib was observed to have comparable binding EC_50_ = 57 nM affinity to the wild-type. We also tested the direct binding of the BID peptides to Mcl-1. In direct binding with Mcl-1 minimal changes in binding affinity were observed. Again FITC-BID-WT showed a high affinity EC_50_ = 98 nM, with EC_50_ = 153 nM for FITC-BID-MM, EC_50_ = 129 nM for FITC-BID-DM and EC_50_ = 84 nM for FITC-BID-Aib.

The subtle differences in profile between the competition and direct titrations are likely to arise as a consequence of interaction of the labelled peptide being displaced with itself and with other components present in the equilibrium (*i.e.* the titrant peptide); this is not uncommon for FA competition assays for which an analytical solution that distinguishes between specific and non-specific effects is not viable.^46^

In summary, only subtle differences were observed for the inhibition of pro-apoptotic Bcl-x_L_ and Mcl-1 interactions using the pan-Bcl-2 binding BID sequence upon introduction of a hydrocarbon constraint. In contrast, a significant loss of inhibition of pro-apoptotic Bcl-x_L_ and Mcl-1 interactions using the pan-Bcl-2 binding BIM sequence upon introduction of a hydrocarbon constraint was observed. The results also demonstrate that constraining a peptide with a hydrocarbon linker has differential effects upon the selectivity and specificity profile of Bcl-2 family recognition properties. Indeed, Walensky and co-workers have noted that constraining peptides can give target dependent results.^5,47^

### Structural properties of constrained peptides bound to Bcl-2 family proteins

**Fig. 6:**
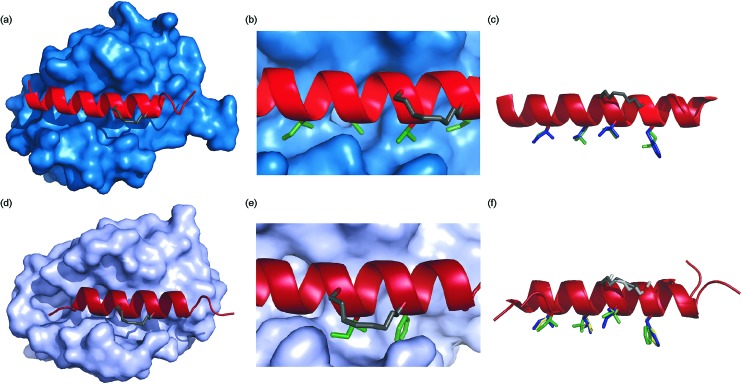
Crystal structures of constrained peptide/protein interactions (a) Mcl-1/BID-MM (PDB entry 5C3F) (b) enlargement of Mcl-1/BID-MM interface illustrating *cis* double bond and key side chains (c) overlay of BID-MM and Mcl-1/BIM-WT from PDB ID: ; 2NL9 illustrating similarity of binding interface. Side chains from BID-MM are shown in green, with BIM-WT side chains in blue. (d) Bcl-x_L_/BIM-MM (PDB entry ; 5C3G) (e) enlargement of Bcl-xL/BIM-MM interface illustrating *cis* double bond and key side chains (f) overlay of BIM-MM, BIM-WT (from PDB ID: ; 3FDL) and BIM-DM (from PDB ID: ; 2YQ6) illustrating similarity of binding interface side chains from BIM-MM are shown in green, with BIM-WT side chains in blue and BIM-DM side chains in yellow.

To ascertain whether the constraint alters the recognition of the hot-spot residues within the peptide, BID-MM was co-crystallised with Mcl-1. The resulting structure is the first BID/Mcl-1 structure reported and the highest resolution structure seen thus far with a hydrocarbon constrained peptide/target complex, at 1.43 Å (PDB entry 5C3F). When in complex with Mcl-1, the hydrocarbon constraint in BID-MM is positioned away from the protein surface, hence not contributing directly to binding (Fig. 6a and b). The double bond within the constraint is in a *cis* configuration, with no electron density to suggest that it exists in a *trans* configuration (Fig. 6b, ESI Fig. S11†). BID-MM binds analogously to BIM-WT in such a way that the constraint doesn't affect the positioning of the hot-spot residues (Fig. 6c). The salt bridge between the conserved BH3 aspartic acid and a conserved arginine in the BH1 domain of Mcl-1 is also retained.^48^

To ascertain whether the constraint also alters the recognition of the hot-spot residues within the BIM peptide, BIM-MM was co-crystallised with Bcl-x_L_ and the structure was refined to 2.45 Å (PDB entry ; 5C3G). The hydrocarbon constraint in BIM-MM is also positioned away from the protein surface and in a *cis* conformation (Fig. 6d, ESI Fig. S12†). The constraint itself projects out into solvent and does not contribute to the interaction between the peptide and Bcl-x_L_ (Fig. 6e). When compared to the structure of Bcl-x_L_ bound to BIM-WT and BIM-DM,^43^ there are no significant differences in the orientation of hot-spot side chains or registry of the peptide (Fig. 6f) – therefore the reason for the drop in potency is not explained by the static structure of the bound complex.

### Kinetics of binding

**Fig. 7:**
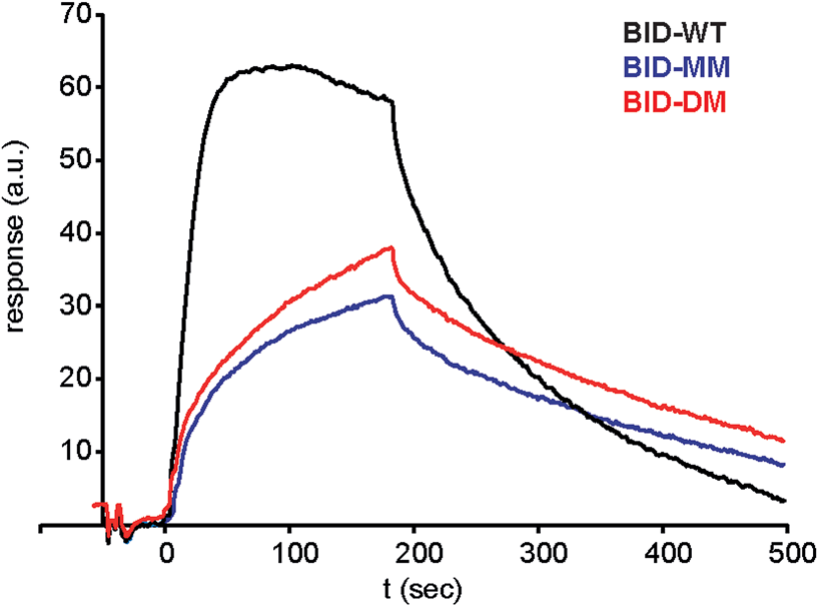
Surface plasmon resonance (SPR) sensorgram of BIM peptides at 100 nM interacting with immobilised Bcl-x_L_.

Given that the constrained peptides adopted a more helical conformation but did not exhibit increased potency, we expanded our study to include a more detailed investigation of the kinetics and thermodynamics of binding. To investigate how the hydrocarbon constraint affects binding kinetics, we established a surface plasmon resonance (SPR) based assay. Bcl-x_L_ was immobilised on a sensor chip *via* its N-terminal His-SUMO tag. BID-WT and BID-MM were then flowed over the immobilised protein. The *K*_d_, on and off rates were calculated from the data fitted to a 1 : 1 model using an average of at least 3 sets of data recorded at various concentrations, all with chi-squared values of below 10% of the *R*_max_ (Table 3, Fig. 7, ESI Fig. S13†). The affinity of BID-MM was slightly lower than with BID-WT, at 107 nM compared to 57 nM respectively. These values are within one order of magnitude to those observed in direct FA, which is reasonable given the presence of a FITC fluorophore for the latter. The on rate was altered, being 10 times slower for BID-MM (3.1 × 10^4^ M s^–1^ compared to 1.8 × 10^5^ M s^–1^), whilst the off-rate was also diminished by nearly one order of magnitude (3.0 × 10^–3^ s^–1^ compared to 1.0 × 10^–2^ s^–1^ with BID-WT). These experiments were repeated for the BIM series of peptides. The on rate was also altered in this series of peptides, being 10 times slower with BIM-MM and BIM-DM (2.08 × 10^4^ M s^–1^ compared to 2.3 × 10^5^ M s^–1^ for BIM-WT).

**Table 3:** SPR analysis of constrained peptides binding to Bcl-x_L_

BH3 peptide	Bcl-x_L_ *k*_on_	Bcl-x_L_ *k*_off_	Bcl-x_L_ *K*_d_
BID-WT	1.8 × 10^6^ M s^–1^ ± 0.42 × 10^6^ M s^–1^	1.0 × 10^–2^ s^–1^ ± 0.18 × 10^–2^ s^–1^	57 nM ± 16 nM
BID-MM	3.1 × 10^4^ M s^–1^ ± 0.9 × 10^4^ M s^–1^	3.0 × 10^–3^ s^–1^ ± 0.7 × 10^–3^ s^–1^	107 nM ± 29 nM
BID-DM	1.75 × 10^4^ M s^–1^ ± 0.3 × 10^4^ M s^–1^	3.0 × 10^–3^ s^–1^ ± 1.0 × 10^–3^ s^–1^	178 nM ± 52 nM
BIM-WT	2.3 × 10^5^ M s^–1^ ± 6.3 × 10 ^4^ M s^–1^	9 × 10^–3^ s^–1^ ± 0.9 × 10^–3^ s^–1^	44 nM ± 17 nM
BIM-MM	2.08 × 10^4^ M s^–1^ ± 0.7 × 10^4^ M s^–1^	8 × 10 ^–3^ s^–1^ ± 0.8 × 10 ^–3^ s^–1^	460 nM ± 232 nM
BIM-DM	2.09 × 10^4^ M s^–1^ ± 1.8 × 10^4^ M s^–1^	9.7 × 10^–3^ s^–1^ ± 0.9 × 10^–3^ s^–1^	1.6 μM ± 0.8 μM

### Thermodynamics of binding

**Fig. 8:**
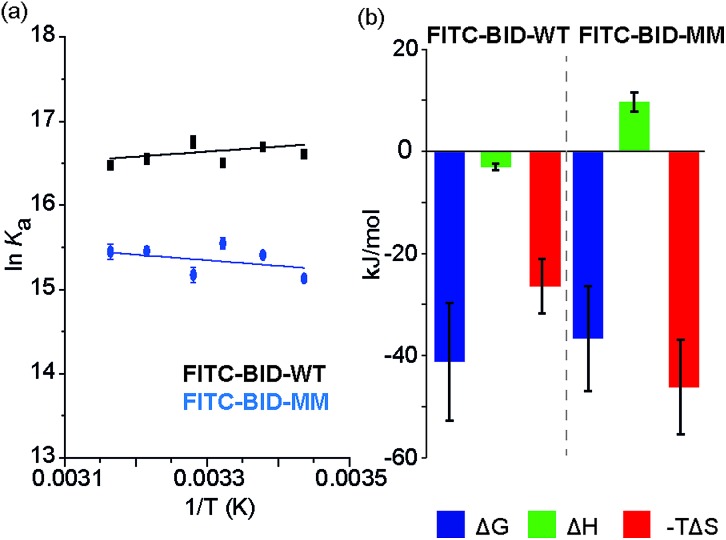
(a) van't Hoff analysis of FITC-BID-WT and FITC-BID-MM interacting with Bcl-x_L_, (50 mM HEPES pH 7.5, 200 mM NaCl, 0.05% Tween 20, recorded at 18-43 °C). (b) Thermodynamic signatures of WT and MM BID peptides binding to Bcl-x_L_.

We investigated the thermodynamics of binding using Van't Hoff analyses of fluorescence anisotropy direct binding experiments (Fig. 8). The full titration was recorded in triplicate at increasing temperatures from 18 °C to 43 °C. The data could be fit to a 1 : 1 model permitting determination of *K*_d_ (see: ESI Fig. S14 and S15†). The resultant data were plotted for 1/*T* against ln *K*_a_ (Fig. 8) and used to estimate Δ*H* and Δ*S*. Comparing the contributions of enthalpy and entropy to the interaction (Fig. 8b), binding of FITC-BID-MM to Bcl-x_L_ is entropically more favourable than binding of FITC-BID-WT to Bcl-x_L_, supporting the expectation that the entropic cost of binding is reduced upon constraining. This is compensated for by an opposing change to the enthalpic contributing to binding. This behaviour is entirely consistent with many co-operative biomolecular protein–ligand interactions.^49^

## Discussion

**Fig. 9:**
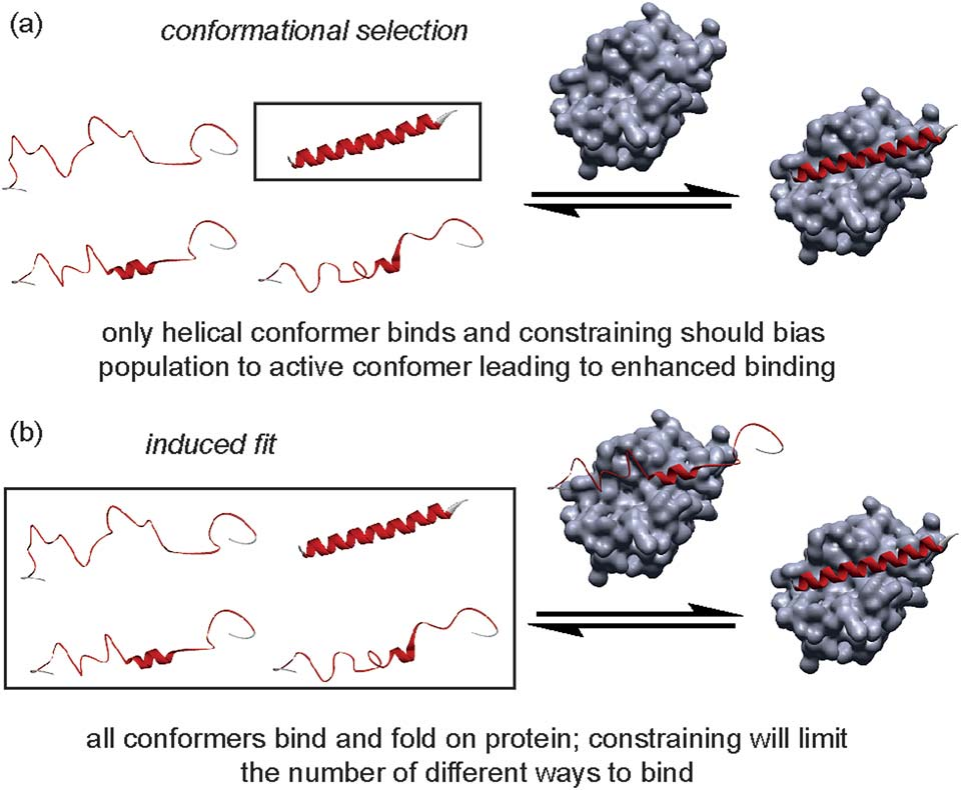
Schematics depicting different binding mechanisms available for Bcl-2 family/BH3 helix mediated PPIs.

The folding of a polypeptide into an ordered conformation is dependent upon enthalpic contributions from backbone or side chain hydrogen-bonding,^50^ other electrostatic forces (*e.g.* π–π stacking) and the hydrophobic effect counterbalanced against entropic contributions from fixing chain entropy and solvent reorganisation. These two large opposing thermodynamic values result in only marginal stability of the folded form^45^ whereby the magnitude of any single non-covalent interaction may be comparable in magnitude to the overall Δ*G* of folding. Outside the context of a folded tertiary structure, in solution, short α-helical peptides usually exist in a random coil or intrinsically disordered conformation.^45^ α-Helix formation occurs through initial nucleation in a region of the helix, followed by propagation throughout the entire sequence (the Zimm–Bragg theory);^45,51^ for short peptides, the entropic cost of helix nucleation is not offset by favourable enthalpic gains from newly formed non-covalent interactions during nucleation and propagation.^52^ In the simplest sense, by introducing a constraint into a peptide that biases its structure towards its bioactive helical conformation, the “cost” of nucleation should have already been paid and for subsequent interaction with a partner protein, the overall affinity for the target of the peptide should increase. An upper limit on what might be achieved in terms of potency enhancement can be estimated to match closely the difference in stability (ΔΔ*G*) between the constrained and non-constrained sequences (which we estimate here to be <3 kJ mol^–1^). However, this represents an oversimplification; the constraint on the peptide may make productive non-covalent interactions^47^ or introduce steric clashes^53^ with the partner protein. Moreover, the mechanism of folding (Fig. 9) has recently been proposed to influence binding affinity; for the interaction between Mcl-1 and PUMA,^54,55^ mutations within PUMA modulated the residual helicity, but not the affinity of the peptides towards Mcl-1.^54,55^ Furthermore, more helical PUMA variants were shown to bind and unbind from Mcl-1 more slowly. Conformational selection suggests that the target protein only recognises the peptide when it briefly forms a helical structure, whereas an induced fit mechanism of binding occurs where an unstructured peptide is recognised and folds on the binding surface.^56^ For conformational selection a greater proportion of the bioactive conformer might reasonably be expected to result in a more efficient interaction with its target whereas for induced-fit, the constrained (pre-organised) peptide might have more limited “ways to bind”^57^ or may interfere with the binding pathway.

Our SPR studies reveal in all cases a significant decrease in on and off rates for binding to Bcl-x_L_ when either BID or BIM are constrained. This (i) is consistent with an induced fit “bind-and-fold” mechanism, (ii) suggests this binding mechanism is general to Bcl-2 family interactions (and not limited to PUMA/Mcl-1) and (iii) suggests for the first time that covalently constraining a peptide can have similar consequences to binding kinetics as are observed when non-covalent forces are used to bias conformation. However, the binding mechanism need not influence binding thermodynamics; our Van't Hoff analyses indicate that constraining leads to more entropically favourable binding but this is offset by an opposing change in enthalpy. Okamoto and colleagues interpret the reduced binding affinity of BIM peptides constrained using α,α′-disubstituted amino acids towards Bcl-x_L_ and Mcl-1 as arising due to loss of favourable intramolecular non-covalent side-chain interactions within the peptide upon binding Bcl-x_L_ or Mcl-1. However, these “lost” interactions are not needed in the constrained peptide and make no direct favourable enthalpic contacts with the target protein. With our X-ray crystallographic analysis suggesting that the bound states of the constrained and unconstrained systems studied here are similar, the thermodynamic binding behaviour can be better accounted for by considering the unbound peptides rather than the protein–peptide complex. While the constrained peptide will have lower entropy as a result of pre-organisation, the backbone hydrogen-bonding is also already present giving a more enthalpically favourable starting condition. Conversely the unconstrained more disordered system has a higher entropy in the unbound state, however this is offset by the absence of favourable backbone hydrogen bonding interactions which are only gained on formation of the unconstrained-ligand/protein complex. If these entropic and enthalpic differences in the unbound states balance then their free energies will be identical and, with nearly identical bound states, the overall binding energies will also be similar. Thus, making an assumption that constraining a peptide changes the energetic state relative to the WT sequence is not valid as the sequences are different. Although the helical state is preferred in the constrained system this can be considered to be due to an increase in the energy of the unfolded state rather than due to absolute preorganization of the helical conformation relative to the unconstrained sequence.

## Conclusions

In summary, we have illustrated that α-pentylglycine represents a powerful amino acid with which to introduce a hydrocarbon constraint, with the advantage of being easier to synthesise and couple during peptide synthesis. Crystal structures of Mcl-1/BID-MM and Bcl-x_L_/BIM-MM revealed that the constrained peptides adopt comparable binding conformations when compared to available structures with wild type sequences and/or peptides constrained with the α,α′-disubstituted alkenyl amino acids. Furthermore our structures confirm that the constraints point away from the BH3 binding cleft and do not introduce steric constraints or new non-covalent interactions with the anti-apoptotic partner. Additional biophysical studies using SPR and van't Hoff analyses are consistent with an induced fit binding mechanism and enthalpy–entropy compensation. This contradicts the hypothesis that introduction of a constraint to pre-organise a peptide should enhance its protein binding affinity. More generally, these studies underscore the need to probe carefully the effects of pre-organisation on protein–ligand interactions – where the constraint is introduced on a ligand that can readily adopt its bioactive conformation and already has optimal binding interactions, it is unlikely to positively influence the binding potency.

## References

[cit1] Arkin M. R., Tang Y., Wells J. A. (2014). Chem. Biol..

[cit2] Azzarito V., Long K., Murphy N. S., Wilson A. J. (2013). Nat. Chem..

[cit3] Chu Q., Moellering R. E., Hilinski G. J., Kim Y.-W., Grossmann T. N., Yeh J. T. H., Verdine G. L. (2015). Med. Chem. Commun..

[cit4] Walensky L. D., Bird G. H. (2014). J. Med. Chem..

[cit5] Bird G. H., Gavathiotis E., LaBelle J. L., Katz S. G., Walensky L. D. (2014). ACS Chem. Biol..

[cit6] Lau Y. H., de Andrade P., Wu Y., Spring D. R. (2015). Chem. Soc. Rev..

[cit7] Dhar A., Mallick S., Ghosh P., Maiti A., Ahmed I., Bhattacharya S., Mandal T., Manna A., Roy K., Singh S., Nayak D. K., Wilder P. T., Markowitz J., Weber D., Ghosh M. K., Chattopadhyay S., Guha R., Konar A., Bandyopadhyay S., Roy S. (2014). Biopolymers.

[cit8] Lee E. F., Smith B. J., Horne W. S., Mayer K. N., Evangelista M., Colman P. M., Gellman S. H., Fairlie W. D. (2011). ChemBioChem.

[cit9] Fremaux J., Mauran L., Pulka-Ziach K., Kauffmann B., Odaert B., Guichard G. (2015). Angew. Chem., Int. Ed..

[cit10] Leduc A.-M., Trent J. O., Wittliff J. L., Bramlett K. S., Briggs S. L., Chirgadze N. Y., Wang Y., Burris T. P., Spatola A. F. (2003). Proc. Natl. Acad. Sci. U. S. A..

[cit11] Harrison R. S., Shepherd N. E., Hoang H. N., Ruiz-Gómez G., Hill T. A., Driver R. W., Desai V. S., Young P. R., Abbenante G., Fairlie D. P. (2010). Proc. Natl. Acad. Sci. U. S. A..

[cit12] Sia S. K., Carr P. A., Cochran A. G., Malashkevich V. N., Kim P. S. (2002). Proc. Natl. Acad. Sci. U. S. A..

[cit13] Xie X., Piao L., Bullock B. N., Smith A., Su T., Zhang M., Teknos T. N., Arora P. S., Pan Q. (2014). Oncogene.

[cit14] Kushal S., Lao B. B., Henchey L. K., Dubey R., Mesallati H., Traaseth N. J., Olenyuk B. Z., Arora P. S. (2013). Proc. Natl. Acad. Sci. U. S. A..

[cit15] Walensky L. D., Kung A. L., Escher I., Malia T. J., Barbuto S., Wright R. D., Wagner G., Verdine G. L., Korsmeyer S. J. (2004). Science.

[cit16] Lama D., Quah S. T., Verma C. S., Lakshminarayanan R., Beuerman R. W., Lane D. P., Brown C. J. (2013). Sci. Rep..

[cit17] Frank A. O., Vangamudi B., Feldkamp M. D., Souza-Fagundes E. M., Luzwick J. W., Cortez D., Olejniczak E. T., Waterson A. G., Rossanese O. W., Chazin W. J., Fesik S. W. (2014). J. Med. Chem..

[cit18] Spiegel J., Cromm P. M., Itzen A., Goody R. S., Grossmann T. N., Waldmann H. (2014). Angew. Chem., Int. Ed..

[cit19] Cromm P. M., Spiegel J., Grossmann T. N. (2015). ACS Chem. Biol..

[cit20] Checco J. W., Lee E. F., Evangelista M., Sleebs N. J., Rogers K., Pettikiriarachchi A., Kershaw N. J., Eddinger G. A., Belair D. G., Wilson J. L., Eller C. H., Raines R. T., Murphy W. L., Smith B. J., Gellman S. H., Fairlie W. D. (2015). J. Am. Chem. Soc..

[cit21] Torres O., Yüksel D., Bernardina M., Kumar K., Bong D. (2008). ChemBioChem.

[cit22] Jo H., Meinhardt N., Wu Y., Kulkarni S., Hu X., Low K. E., Davies P. L., DeGrado W. F., Greenbaum D. C. (2012). J. Am. Chem. Soc..

[cit23] Kawamoto S. A., Coleska A., Ran X., Yi H., Yang C.-Y., Wang S. (2012). J. Med. Chem..

[cit24] Muppidi A., Doi K., Edwardraja S., Drake E. J., Gulick A. M., Wang H.-G., Lin Q. (2012). J. Am. Chem. Soc..

[cit25] Lau Y. H., de Andrade P., Quah S.-T., Rossmann M., Laraia L., Skold N., Sum T. J., Rowling P. J. E., Joseph T. L., Verma C., Hyvonen M., Itzhaki L. S., Venkitaraman A. R., Brown C. J., Lane D. P., Spring D. R. (2014). Chem. Sci..

[cit26] Nogami K., Takahama K., Okushima A., Oyoshi T., Fujimoto K., Inouye M. (2014). ChemBioChem.

[cit27] Haney C. M., Horne W. S. (2015). Org. Biomol. Chem..

[cit28] Rogers J. M., Wong C. T., Clarke J. (2014). J. Am. Chem. Soc..

[cit29] O'Neil K., DeGrado W. (1990). Science.

[cit30] Aurora R., Rosee G. D. (1998). Protein Sci..

[cit31] Schafmeister C. E., Po J., Verdine G. L. (2000). J. Am. Chem. Soc..

[cit32] Kim Y. W., Kutchukian P. S., Verdine G. L. (2010). Org. Lett..

[cit33] Blackwell H. E., Grubbs R. H. (1998). Angew. Chem., Int. Ed..

[cit34] Yeo D. J., Warriner S. L., Wilson A. J. (2013). Chem. Commun..

[cit35] Douse C. H., Maas S. J., Thomas J. C., Garnett J. A., Sun Y., Cota E., Tate E. W. (2014). ACS Chem. Biol..

[cit36] Moldoveanu T., Follis A. V., Kriwacki R. W., Green D. R. (2014). Trends Biochem. Sci..

[cit37] Czabotar P. E., Lessene G., Strasser A., Adams J. M. (2013). Nat. Rev. Mol. Cell Biol..

[cit38] Lessene G., Czabotar P. E., Colman P. M. (2008). Nat. Rev. Drug Discovery.

[cit39] Chen L., Willis S. N., Wei A., Smith B. J., Fletcher J. I., Hinds M. G., Colman P. M., Day C. L., Adams J. M., Huang D. C. S. (2005). Mol. Cell.

[cit40] Certo M., Moore V. D. G., Nishino M., Wei G., Korsmeyer S., Armstrong S. A., Letai A. (2006). Cancer Cell.

[cit41] Walensky L. D., Pitter K., Morash J., Oh K. J., Barbuto S., Fisher J., Smith E., Verdine G. L., Korsmeyer S. J. (2006). Mol. Cell.

[cit42] Belokon Y. N., Bulychev A. G., Vitt S. V., Struchkov Y. T., Batsanov A. S., Timofeeva T. V., Tsyryapkin V. A., Ryzhov M. G., Lysova L. A., Bakhmutov V. I., Belikov V. M. (1985). J. Am. Chem. Soc..

[cit43] Okamoto T., Zobel K., Fedorova A., Quan C., Yang H., Fairbrother W. J., Huang D. C. S., Smith B. J., Deshayes K., Czabotar P. E. (2013). ACS Chem. Biol..

[cit44] LaBelle J. L., Katz S. G., Bird G. H., Gavathiotis E., Stewart M. L., Lawrence C., Fisher J. K., Godes M., Pitter K., Kung A. L., Walensky L. D. (2012). J. Clin. Invest..

[cit45] Scholtz J. M., Baldwin R. L. (1992). Annu. Rev. Biophys. Biomol. Struct..

[cit46] Roehrl M. H. A., Wang J. Y., Wagner G. (2004). Biochemistry.

[cit47] Stewart M. L., Fire E., Keating A. E., Walensky L. D. (2010). Nat. Chem. Biol..

[cit48] Czabotar P. E., lee E. F., Delft M. F. v., Day C. L., Smith B. J., Huang D. C. S., Fairlie W. D., Hinds M. G., Colman P. M. (2007). Proc. Natl. Acad. Sci. U. S. A..

[cit49] Hunter C. A., Tomas S. (2003). Chem. Biol..

[cit50] Myers J. K., Pace C. N. (1996). Biophys. J..

[cit51] Zimm B. H., Bragg J. K. (1959). J. Chem. Phys..

[cit52] Miller S. E., Watkins A. M., Kallenbach N. R., Arora P. S. (2014). Proc. Natl. Acad. Sci. U. S. A..

[cit53] Phillips C., Roberts L. R., Schade M., Bazin R., Bent A., Davies N. L., Moore R., Pannifer A. D., Pickford A. R., Prior S. H., Read C. M., Scott A., Brown D. G., Xu B., Irving S. L. (2011). J. Am. Chem. Soc..

[cit54] Rogers J. M., Oleinikovas V., Shammas S. L., Wong C. T., de Sancho D., Baker C. M., Clarke J. (2014). Proc. Natl. Acad. Sci. U. S. A..

[cit55] Rogers J. M., Wong C. T., Clarke J. (2014). J. Am. Chem. Soc..

[cit56] Csermely P., Palotai R., Nussinov R. (2010). Trends Biochem. Sci..

[cit57] Williams D. H., Stephens E., O'Brien D. P., Zhou M. (2004). Angew. Chem., Int. Ed..

