# Supplementary material for: Hydrocarbon constrained peptides – understanding preorganisation and binding affinity
Source: Chem Sci. 2016 Feb 29;7(6):3694–702. doi: 10.1039/c5sc04048e (PMC5618334; doi:10.1039/c5sc04048e)
Supplement: Supplementary file 1 [file SC-007-C5SC04048E-s001.pdf]

Electronic Supplementary Material (ESI) for Chemical Science.
This journal is © The Royal Society of Chemistry 2016

Hydrocarbon Constrained Peptides – Understanding Preorganization and Binding Affinity

Jennifer A. Miles,[1,2] David J. Yeo,[1,2] Philip Rowell,[2,3] Silvia Rodriguez-Marin,[1,-2] Christopher M. Pask,[1] Stuart L. Warriner,[1,2] Thomas A. Edwards,[2,3]* Andrew J. Wilson[1,2]*

[1]School of Chemistry, [2]Astbury Centre for Structural Molecular Biology, [3]School of Molecular and Cellular Biology, University of Leeds, Woodhouse Lane, Leeds LS2 9JT, e-mail: A.J.Wilson@leeds.ac.uk, T.A.Edwards@leeds.ac.uk

SUPPORTING INFORMATION

Contents

Alkylated Ni-Gly-BPB Complex 2
(*S*)-2-((((9H-fluoren-9-yl)methoxy)carbonyl)amino)-2-methylhept-6-enoic acid 2
Procedures for peptide synthesis 3
Synthesis of BID peptides 3
General Regards 3
Methods for Manual Fmoc Solid Phase Peptide Synthesis 3
Cycles for automated peptide synthesis 5
Peptide Purification 5
Synthesis of FITC-labelled BID peptides 6
Synthesis of BIM peptides 6
HPLC, ESI-MS and LC-MS Data for peptides 7
Circular Dichroism 12
Overexpression and Purification of Bcl-x_L and Mcl-1 13
Fluorescence Anisotropy 13
BODIPY-BAK/Bcl-x_L competition assays 13
FITC-NOXA-B/Mcl-1 competition assays 13
FITC-BID/Mcl-1 and FITC-BID/Bcl-x_L direct binding assays 14
Processing of fluorescence anisotropy data 14
Supplementary Fluorescence Anisotropy assays 15
X-ray Data Collection and Refinement Statistics 20
Electron density of stapled regions 21
SPR of BIM series of peptides 22
Van’t Hoff analysis of direct binding van't Hoff analysis of fluorescence anisotropy data 22
References 25

Alkylated Ni-Gly-BPB Complex

(*S*)-2-((((9H-fluoren-9-yl)methoxy)carbonyl)amino)-2-methylhept-6-enoic acid

The amino acid was synthesized according to the method previously described.[1] Crystallisation of the alkylated nickel (II) Schiff base complex from a toluene/pentane solution prior to isolation and Fmoc protection of the amino acid confirmed the stereochemistry (see below for details of the single crystal diffraction study).

Measurements were carried out at 100K on an Agilent Supernova diffractometer equipped with an Atlas CCD detector and connected to an Oxford Cryostream low temperature device using graphite monochromated Cu K_α radiation (λ = 1.54184 Å) from a Microfocus Nova X-ray source. The structure was solved by direct methods using SHELXS[2] and refined using SHELX97. The compound crystallised as orange plates in a monoclinic cell and was solved in the $P2_1$ space group, with two molecules in the asymmetric unit. Most non-hydrogen atoms were located in the Fourier Map and refined anisotropically. There was disorder around the aromatic ring of one of the benzyl groups (C34 to C39). This was refined as two rings each with 50% occupancy. C34, C35A to C39A and C35B to C39B were refined isotropically. All hydrogen atoms were placed in calculated positions and refined isotropically using a "riding model". An orange plate of approximate size 0.15 x 0.07 x 0.04 mm was used for data collection. Multiscan acquisition. θ range = 7.6 ≤ θ ≤ 132.2°, formula = $C_{32}H_{33}N_3NiO_3$; formula weight = 566.32; a = 11.07980(8)Å, b = 21.99770(13)Å, c = 11.88015(9)Å, β = 102.0025(7); volume = 2832.25(3) Å^3; Z=4; D(calculated): 1.328 g/cm^3; μ = 1.284 mm^{-1}; reflections collected 21899; independent reflections 10002 [R_{int} = 0.0253, R_{sigma} = 0.0327]; observed reflections 6564 [I>2s(I)]; R value = 0.0273, wR_2 = 0.0688 During the data collection and reduction, Friedel pairs were unmerged, allowing determination of the stereochemistry of the complex. Flack parameter,[3] = -0.005(12).

CCDC 1057141 contains the supplementary crystallographic data for this paper. These data can be obtained free of charge from the Cambridge Crystallographic Data Centre via www.ccdc.cam.ac.uk/data_request/cif.

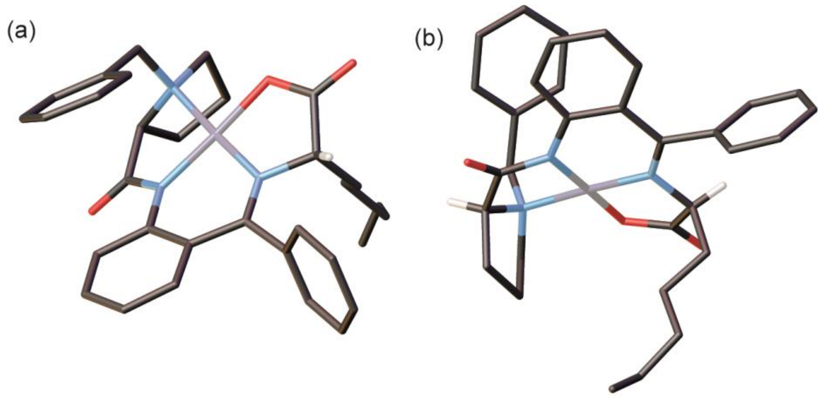

Figure SX. X-ray crystal structure of alkylated complex, confirming the absolute stereochemistry of the alkylation. Hydrogen atoms other than at the α-position have been omitted for clarity. (Colour code: Grey = C, Blue = N, Red = O, Silver = Ni). a) top perspective; b) lower perspective

Procedures for peptide synthesis

Synthesis of BID peptides

Synthesis was as described previously[1]

General Regards

All amino acids and resins were purchased from either Novabiochem (Merck) or Sigma-Aldrich. All amino acids were *N*-Fmoc protected and side chains were protected with Boc (Lys, Trp); *O*^{t}Bu (Asp, Glu, Ser, Thr); Trt (Cys, Asn, Gln); Pbf (Arg). Synthesis of peptides was performed either manually using fritted SPE reservoirs and vacuum filtration tank or by the use of a microwave assisted automated peptide synthesiser (CEM Liberty). DMF used in peptide synthesis was of ACS grade from Sigma-Aldrich.

Methods for Manual Fmoc Solid Phase Peptide Synthesis

Method A: Resin Swelling

The required quantity of resin was placed in a fritted empty SPE tube and CH_2Cl_2 (2 ml) was added and the resin was agitated on a Stuart Rotator-SB2 for 2 h to allow swelling of the resin.

Method B: Deprotection of N-Fmoc protecting groups

N-terminal Fmoc protecting groups were removed by the addition of 20% piperidine: DMF (5 × 2 mL × 2 min), followed by rinsing the resin with DMF (5 × 2 mL × 2 min). Successful deprotection was determined by a positive colour test (Methods C & D).

Method C: Kaiser Test[4]

The Kaiser Test was employed for the determination of the successful coupling or deprotection for most of the residues. A small number of resin beads were rinsed in ethanol and placed in a vial, followed by the addition of two drops of each of the three solutions in the following order:

1) Ninhydrin (5% w/v) in ethanol;

2) Phenol (80% w/v) in ethanol;

3) 1 mM $KCN_{(aq.)}$ in pyridine (2% v/v).

The solution was then heated to ca. 150 °C for 1 min. A successful coupling gave no change in the colour of the beads, whereas bright blue beads illustrate a successful deprotection. This colour test was useful for the identification of free primary amines, however inconclusive results are obtained for Asp, Ser, Pro and Asn residues.

Method D: Chloranil Test[5]

The chloranil test was also employed for the determination of successful couplings or deprotections of some residues. A small number of beads were rinsed in ethanol and placed in a vial, followed by the addition of two drops of each of the two solutions in the following order:

1) Acetaldehyde (2% v/v) in DMF;

2) *p*-Chloranil (2% w/v) in DMF.

The solution was left at rt for 5 min. No change in colour of the beads showed a negative result, whereas the change of bead colour to pale green/bright blue illustrated a successful deprotection.

This test was especially useful for Pro residues, where the bead colour became a very bright blue (often after 10 seconds) to show a free secondary amine. However, the length of time (often ~10 min for a primary amine) for the colour change made this colour test less useful than the Kaiser test for the determination of free primary amines.

Method E: Coupling of Amino Acids with Uronium Coupling Reagents

The desired amino acid (5 equiv.), DIPEA (5 equiv.), HOBt (5 equiv.) and Uronium coupling reagent (Either HATU, HBTU or HCTU) (5 equiv.) were dissolved in DMF (2 mL) and added to the resin, followed by agitation for 1 h (2 h for unnatural amino acids). For double couplings, this step was repeated. After removal of the reagents by filtration, the resin was washed with DMF (3 × 2 mL × 2 min) and the success of coupling determined by a negative colour test (Methods C & D). Deprotection of the Fmoc-protected *N*-terminus then followed (Method B).

Method F: Coupling of Cysteine

The amino acid (5 equiv.), 2,4,6-trimethylpyridine (5 equiv.) and HCTU (5 equiv.) were dissolved in 1:1 CH_2Cl_2:DMF (2 mL) and added to the resin, followed by agitation for 1 h. After draining the reagents, the resin was washed with DMF (3 × 2 mL × 2 min) and the success of coupling determined by a negative colour test (Methods C & D).

Method G: N-terminal acetylation

Acetic anhydride (10 equiv.) and DIPEA (10 equiv.) were dissolved in DMF (2 mL) and the solution was transferred to the resin. After 2 h, the resin was drained, washed with DMF (3 × 2 mL × 2 min) and successful capping determined by a negative colour test (Methods C & D).

Method H: N-terminal FITC labelling[6]

Fluorescein isothiocyanate (1.2 equiv.) was dissolved in 12:8:5 Pyridine:DMF:CH_2Cl_2 (2 mL) and the solution transferred to the resin in the dark. After 18 h, the resin was washed with DMF (5 × 2 mL × 2 min) ahead of cleavage and deprotection (Method K). The solvents were of anhydrous grade and the pyridine distilled prior to use.

Method J: On-Resin Olefin Metathesis

After the completed peptide elongation and *N*-terminal acetylation, on-resin olefin metathesis was completed by the preparation of a 10 mM solution of Grubbs First Generation Catalyst in degassed dichloroethane (2 mL), which was added to the resin beads and allowed to agitate gently for 2 h, after which time the metathesis procedure was repeated for another 2 h. *N*-terminally acetylated peptides were then cleaved from the resin (Method K). For FITC labelled peptides, metathesis was performed before labelling (Method H).

Method K: Cleavage and deprotection of Rink Amide MBHA resin

After elongation and *N*-terminal acetylation was complete, the resin was washed with DMF (5 × 2 mL × 2 min), CH_2Cl_2 (5 × 2 mL × 2 min) and then Et_2O (3 × 2 mL × 2 min). Peptides were then simultaneously cleaved and side-chain deprotected with cleavage 'Reagent K' TFA:EDT:Thioanisole:Phenol:H_2O, 82:3:5:5:5 (3 × 2 mL × 1 h). The resin was washed with fresh TFA (2 mL × 2 min) and the solution concentrated *in vacuo*.

The resulting oil was precipitated with ice-cold ether (10 mL) and placed in a centrifuge (3000 rpm × 0.5 min). The supernatants were removed, the precipitate rinsed with ice-cold ether (3 × 10 mL) and dried *in vacuo*.

Cycles for automated peptide synthesis

Peptides that were built on the microwave assisted Liberty CEM Peptide Synthesiser followed this cycle:

Resin Loading

Clean reaction vessel; wash with DMF; wash with CH_2Cl_2; transfer resin to reaction vessel; wash with DMF; wash with CH_2Cl_2; transfer resin to reaction vessel; wash with DMF; wash with CH_2Cl_2; vessel draining.

Deprotection and coupling

Clean resin dip tube, wash with DMF (15 mL), add 20% piperidine in DMF (6 mL), microwave method (30 sec), wash with DMF (15 mL), add 20% piperidine in DMF (6 mL), microwave method (30 sec), wash with DMF (15 mL), clean resin dip tube, wash with DMF (15 mL), add amino acid (2.5 mL), add coupling reagent (1 mL), add activator base (0.5 mL), microwave method (5 min), wash with DMF (15 mL), drain.

For methods that ***did not*** use microwave assistance, the reaction cycle was the same, except the microwave method for deprotection and coupling was replaced by agitation of the resin at rt for 10 min and 90 min respectively.

After the final residue, the resin was ejected from the reaction vessel and cleavage/deprotection was performed manually using Methods K-M.

Peptide Purification

Peptides were purified by preparative scale HPLC using a Jupiter Proteo preparative column (reversed phase) on an increasing gradient of acetonitrile to water (plus 0.1% formic acid v/v in both solvents) at a flow rate of 20 mL min^{-1}. Crude peptides were suspended in either dimethylsulfoxide or 1:1 acetonitrile:water at an approximate concentration of 15 - 20 mg mL^{-1}. Purification runs injected a maximum of 2.5 mL of crude peptide solution and were allowed to run for 35 min, with acetonitrile increasing from 5 to 95%, and the eluent scanned with a diode array at 220, 254, 280 and 490 nm. Fractions were checked by LCMS, concentrated *in vacuo* and lyophilised. For semi-preparative purification, a Jupiter Proteo Semi-Preparative (reversed phase) column was used, with the injection reduced to 250 µL and the flow rate reduced to 3 mL min^{-1}. For peptides with crude UV traces that were unsuitable for collection by chromatogram, purification was performed on a semi-preparative scale using a mass directed chromatography software Masshunter by ChemStation (Agilent). Mass directed chromatography allows the collection of the desired peptide by mass, with the eluent split into an Agilent 6120 Quadrupole LCMS which triggers collection of eluent at a programmed m/z. The

column used with this apparatus is an Agilent XBridge 5 µM 10×50 mm C_{18} semi preparative column. Injections were 250 µL and flow rate 5 mL min^{-1}.

Synthesis of FITC-labelled BID peptides

FITC labelled BID peptides (FITC-BID-WT, FITC-BID-Aib, FITC-BID-MM and FITC-BID-DM) were synthesised on a 0.05 mmol scale using procedures described in detail in the supporting information. The *N*-termini were functionalised with 6-aminohexanoic acid (FITC-BID-WT) or β-alanine (FITC-BID-Aib, FITC-BID-MM, FITC-BID-DM).

Purification of the FITC-BID-WT, FITC-BID-MM and FITC-BID-DM peptides was performed by semi-preparative HPLC as described in further detail in the supporting information using a Jupiter Proteo semi-preparative column with a gradient of 5-40-60-95% acetonitrile (with 0.1% formic acid) at time 0-5-25-30 mins. The peptides generally eluted between 20-22 minutes. FITC-BID-Aib was purified using a mass-directed semi-preparative method on an Agilent XBridge column, using a gradient of 40-60% acetonitrile (with 0.1 % formic acid) over 20 minutes. FITC-BID-Aib eluted at 6 minutes. The purified yields of the peptides were: FITC-BID-WT 6.5 mg (7%); FITC-BID-Aib 9. 9 mg (10 %), FITC-BID-MM 23 mg (21 %) and FITC-BID-DM 9.25 mg (10%).

Synthesis of BIM peptides

The BIM series of peptides (BIM MM and BIM DM) were synthesized on Rink Amide MBHA LL resin (Capacity 0.36 mmol g-1) on a scale of 0.1 (BIM MM) and 0.075 (BIM DM) using the methods described in the supporting information and HCTU as the coupling reagent. The peptides were synthesised on the automated peptide synthesiser without microwave assistance, except for the residues encompassing ^{152}RXIGDX which were coupled manually. The Fmoc groups of ^{153}X and ^{157}X were deprotected with each deprotection cycle lasting 5 min. ^{152}R and ^{158}D were double coupled using 10 equivalents of HATU, with the first coupling proceeding for 1 h and the second coupled overnight. Purification was performed using semi-preparative HPLC with a Jupiter Proteo semi-preparative column on a gradient of 0-35-52-95% acetonitrile (with 0.1% formic acid) at times 0-7-27-31 mins with BIM-MM and BID-DM eluting at 16-18 minutes. The purified yields of peptides were: BIM-MM 9.2 mg (23% based on 40 mg purified) and BIM- DM 21.6 mg (32% of 0.038 mmol purified).

Below are tabulation HRMS data of the peptides that have been synthesised. Peptide identity was confirmed by the inspection of multiple charge states and are quoted as the *monoisotopic* peak for the Expected (Exp^d) and Observed (Obs^d) masses

Unlabelled BID peptides

Peptide	$[M+2H]^{2+}$ Obs[d]	$[M+2H]^{2+}$ Exp[d]	$[M+3H]^{3+}$ Obs[d]	$[M+3H]^{3+}$ Exp[d]
BID WT	1359.7318	1359.7253	906.8313	906.8193
BID-Aib	1337.2347	1337.2328	891.8258	891.8243
BID-DM	1377.2529	1377.2641	918.5114	918.5118
BID-MM	1363.2338	1363.2485	909.1673	909.1673

FITC labelled BID peptides

Peptide	$[M+4H]^{4+}$ Obs[d]	$[M+4H]^{4+}$ Exp[d]	$[M+5H]^{5+}$ Obs[d]	$[M+5H]^{5+}$ Exp[d]
FITC-BID-WT	795.3939	795.3942	636.5157	636.5169
FITC-BID-Aib	773.6356	773.6362	619.1093	619.1105
FITC-BID-DM	793.6513	793.6518	635.1209	635.1230
FITC-BID-MM	786.6422	786.6440	629.5143	629.5166

BIM BH3 peptides

Peptide	$[M+3H]^{3+}$ Obs[d]	$[M+3H]^{3+}$ Exp[d]	$[M+4H]^{4+}$ Obs[d]	$[M+4H]^{4+}$ Exp[d]
BIM WT	894.4736	894.4735	671.1072	671.1069
BIM DM	882.8175	882.8150	662.3624	662.3662
**Peptide**	**$[M+3H]^{3+}$ Obs[d]**	**$[M+3H]^{3+}$ Exp[d]**	**$[M+2H]^{2+}$ Obs[d]**	**$[M+2H]^{2+}$ Exp[d]**
BIM MM	873.4713	873.4712	1309.7027	1309.7032

HPLC, ESI-MS and LC-MS Data for peptides

Data for BID-WT,BID-Aib BID-MM and BID-DM has previously been published[1]; these peptides were resynthesized here and gave comparable data..

Synthesis and purification of (i) BAK and BODIPY-BAK[1] and (ii) FITC-NOXA-B and NOXA B[7] were described previously. These reagents were available from the previous work and were not resynthesized.

BAD was purchased from Peptide and Protein Research Ltd.

Shoulders in some of the LC and HPLC traces are due to cis/trans isomers and where applicable, arises from the use of mixed isomers of FITC for peptide synthesis.

FITC-BID-WT

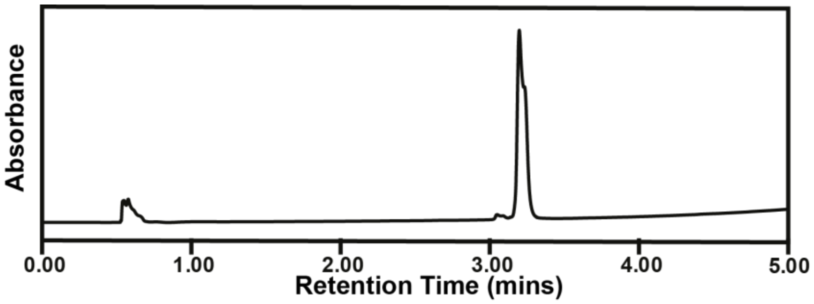

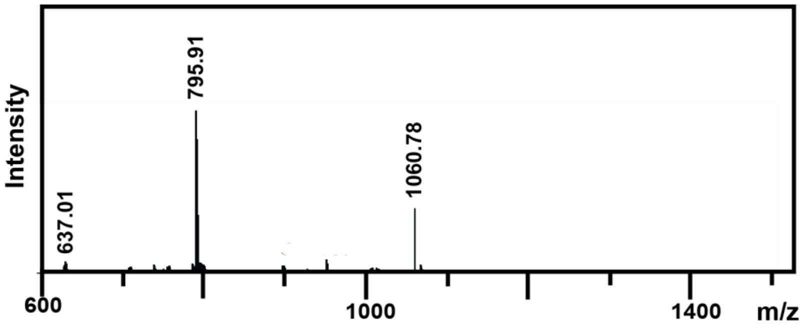

MS data shows $[M+4H]^{4+}$ at 637.01, $[M+3H]^{3+}$ at 795.91 and $[M+2H]^{2+}$ at 1060.78.

FITC-BID-Aib

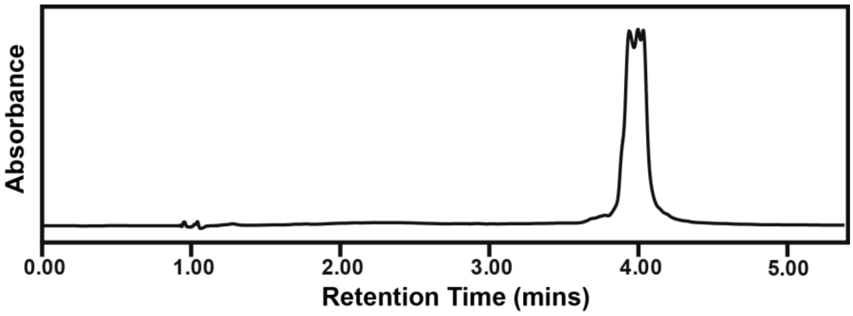

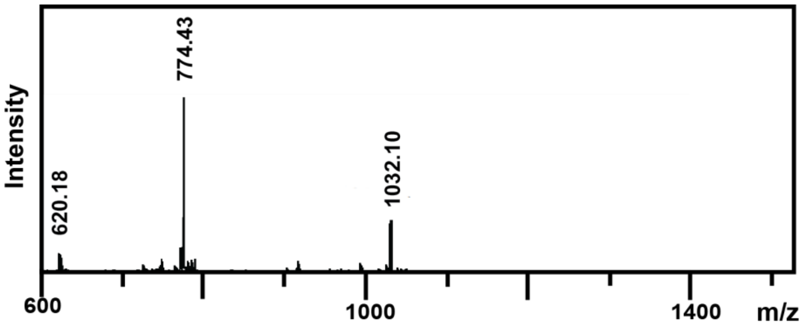

MS data shows $[M+4H]^{4+}$ at 620.18, $[M+3H]^{3+}$ at 774.43 and $[M+2H]^{2+}$ at 1032.10.

FITC-BID-MM

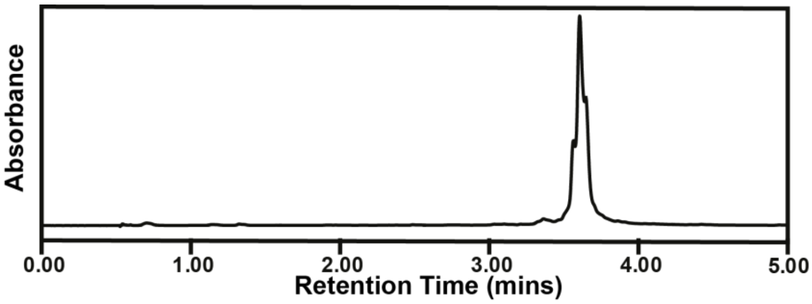

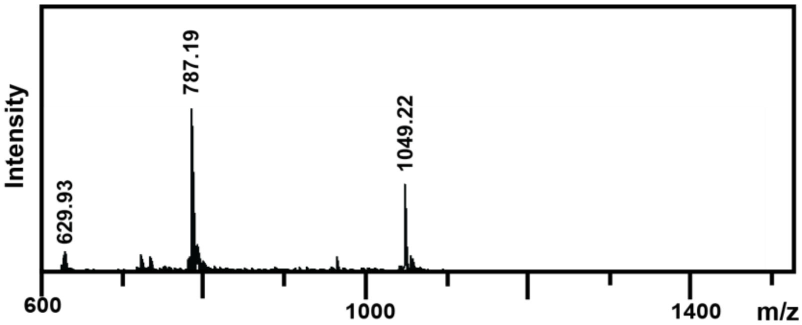

MS data shows the $[M+4H]^{4+}$ at 629.93, $[M+3H]^{3+}$ at 787.19 and $[M+2H]^{2+}$ at 1049.22.

FITC-BID-DM

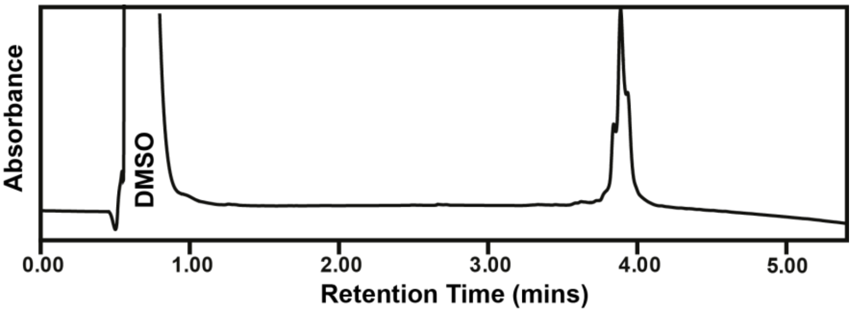

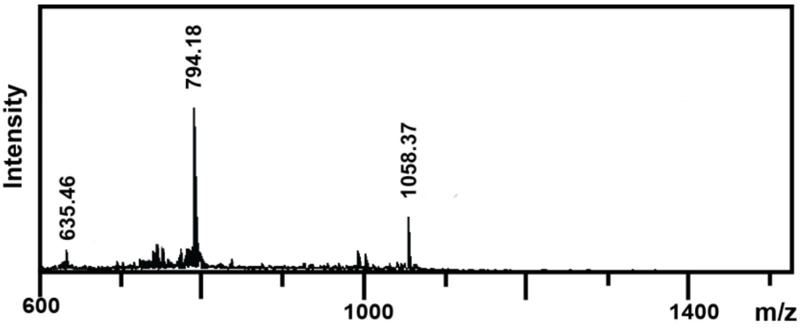

MS data shows the $[M+4H]^{4+}$ at 635.46, $[M+3H]^{3+}$ at 794.18 and $[M+2H]^{2+}$ at 1058.37.

BIM-WT

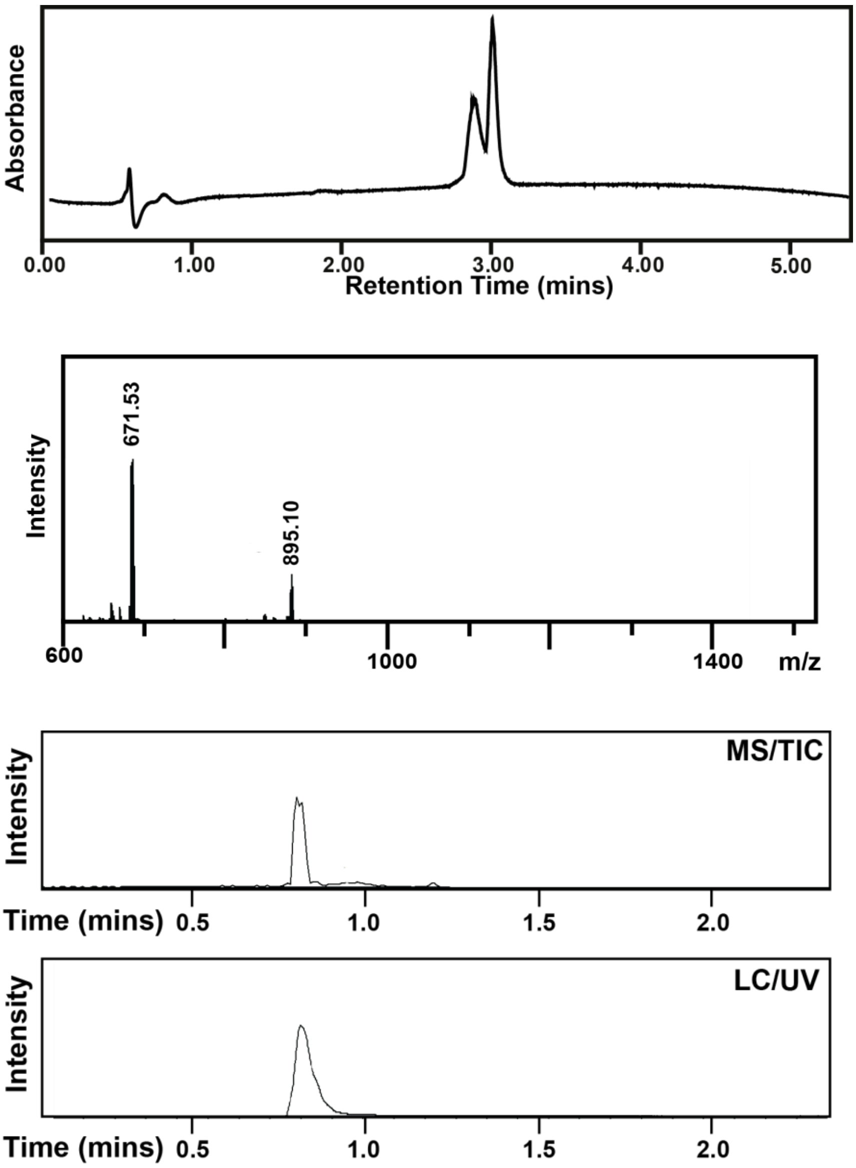

On Analytical HPLC, BIM WT appeared to have two UV peaks, but further analysis with LCMS and altering the HPLC gradient did not separate these peaks out further. Using Loop and LCMS identified only the target peptide with no other species ionising. The MS chromatogram shows $[M+4H]^{4+}$ at 671.53 and $[M+3H]^{3+}$ at 895.10.

BIM-MM

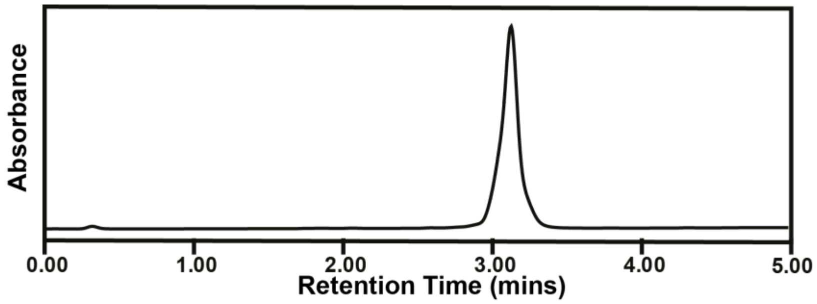

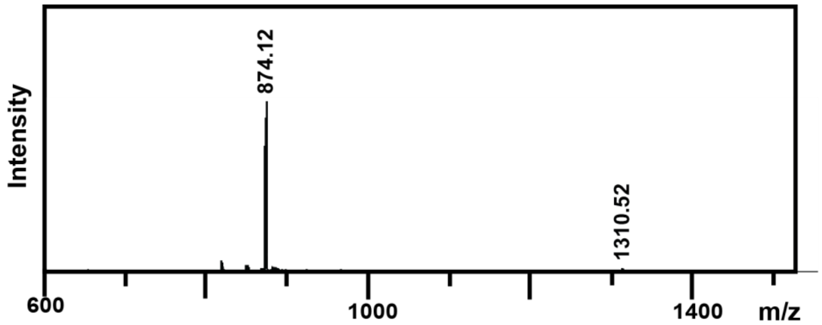

MS Data shows $[M+3H]^{3+}$ at 874.12 and $[M+2H]^{2+}$ at 1310.52.

BIM-DM

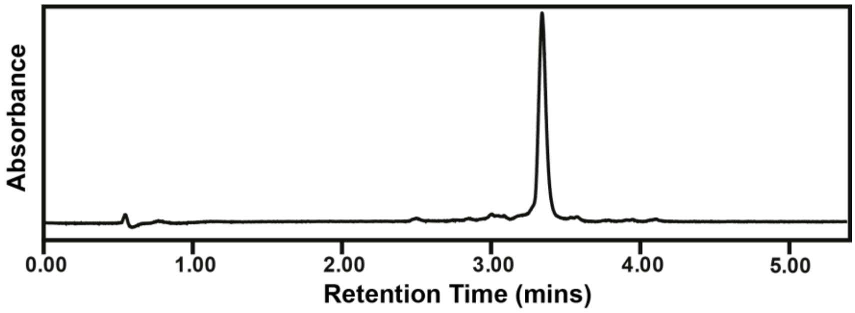

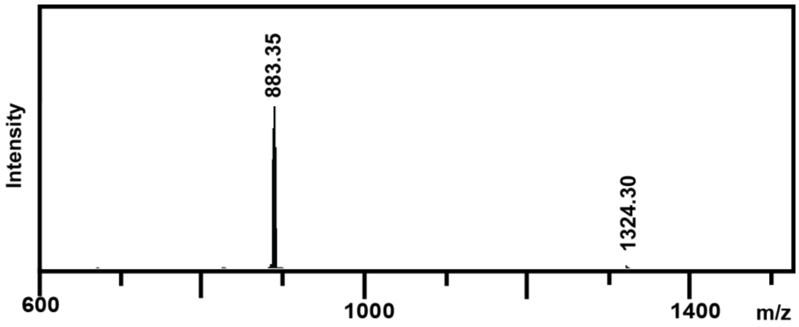

MS Data shows $[M+3H]^{3+}$ at 883.35 and $[M+2H]^{2+}$ at 1324.30.

Circular Dichroism

Circular Dichroism was performed on an Applied Photophysics ChiraScan Apparatus and Software. For each scan, the following parameters were used: 180-260 nm range; point time 1 s; 1 nm per point; step = 1; bandwidth 5 nm; path length 10 mm; temperature 20 °C. Scans were performed in triplicate. Samples were dissolved in 1:4 acetonitrile:50 mM sodium phosphate buffer pH 7.50 to concentrations between 5 - 20 μM. DMSO stocks of peptides were not used for CD due to its high absorbance below 230 nm. The use of organic co-solvent ensures good solubility at higher concentrations used in CD. Moreover it is an acceptable experimental approach for assessing helical propensity in peptides.[8]

The raw circular dichroism data obtained for the peptides was processed by the subtraction of the solvent signal and converted into a mean residue ellipticity:

$$[\theta] = \frac{\theta}{10 \times c \times l}$$

$$[\theta]_{MRE} = \frac{[\theta]}{(R\ -\ 1)}$$

Where θ = circular dichroism at a given wavelength, *c* = molar concentration, l = path length in cm, R = number of residues in the peptide sequence.

$$\Theta = \left(\Theta(0) + \left(\frac{\partial\Theta}{\partial \mathrm{T}}\right)\mathrm{T}\right) \cdot (1 - \frac{\mathrm{x}}{\mathrm{Nr}}))$$

Calculation of % Helicity was performed by the following equation:[9]

Where: Θ = Theoretical MRE for 100% helicity at 222 nm, Θ(0) = Theoretical MRE for 100% helicity at 222 nm at 0 °C = -44000, (∂Θ/∂T) = temperature dependence of infinite helix = +250, T = temperature in °C, x = 3, N_r = number of residues in a peptide.

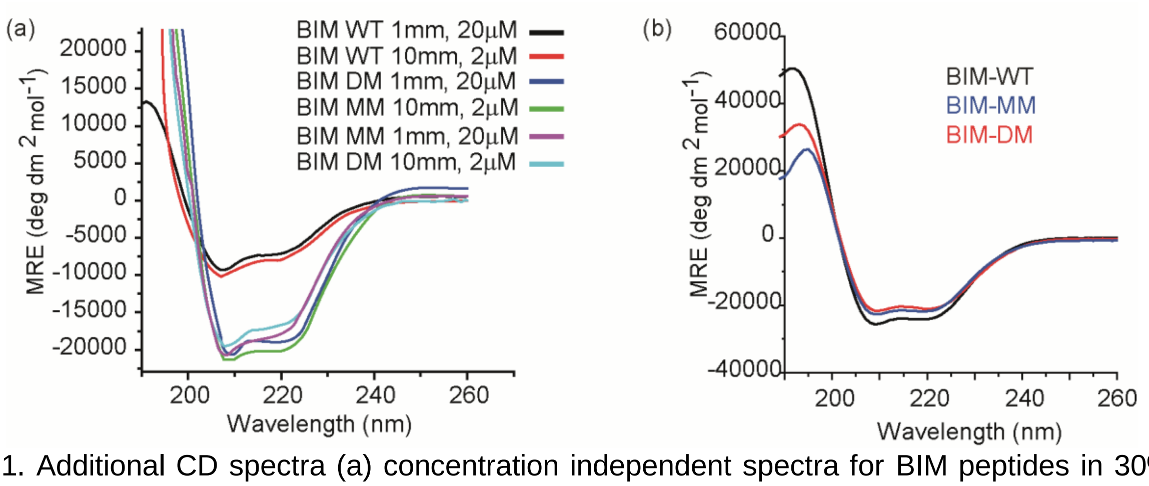

Figure S1. Additional CD spectra (a) concentration independent spectra for BIM peptides in 30% CH_3CN:50 mM sodium phosphate (b) BIM peptides in 30% TFE indicating maximum helicity feasible for these peptide (note: MREs are of the same order of magnitude as the data reported in Figure 2b).

Overexpression and Purification of Bcl-x_L and Mcl-1

The pET28a His-SUMO Mcl-1 (172-327) and pET28a His-SUMO Bcl-x_L (1-198, missing 26-81) constructs were over-expressed in the *E. coli* strain Rosetta 2. Cells were resuspended in 50mM TRIS pH 8.0, 500mM NaCl, 15mM Imidazole and lysed by sonication. The clarified lysate was applied to a 5ml HisTrap column equilibrated with 50mM TRIS pH 8.0, 500mM NaCl, 15mM Imidazole. The HisTrap was then washed with 10 CV of 50mM TRIS pH 8.0, 500mM NaCl, 15mM Imidazole followed by 10 CV 50mM TRIS pH 8.0, 500mM NaCl, 25mM Imidazole. The fusion proteins were eluted in 50mM TRIS pH 8.0, 500mM NaCl, 300mM Imidazole. TheHis-SUMO tag was removed in overnight in dialysis into 50mM TRIS pH 8.0, 250mM NaCl in the presence of Smt3 protease, Ulp1, overnight at 4°C. Uncleaved material and His-SUMO were removed by reapplication of the sample to a HisTrap in 50mM TRIS pH 8.0, 250mM NaCl and the flow through containing Mcl-1 or Bcl-x_L collected. The proteins were then filtered before further purification on a Superdex 75 (GE healthcare) equilibrated in 50mM TRIS pH 8.0, 250mM NaCl, 0.5mM DTT, 2.5% Glycerol. Purified proteins were concentrated and stored at -80°C.

Fluorescence Anisotropy

The assays were performed as detailed in [10, 11].

The buffers used for fluorescence anisotropy were either phosphate buffer (40 mM sodium phosphate, 200 mM sodium chloride, 0.02 mg ml^{-1} bovine serum albumin, pH 7.50) or Tris buffer (50 mM Tris, 140 mM sodium chloride, pH 7.50). Phosphate buffer was used for all competition assays and the direct binding assays for FITC-NOXA-B/Mcl-1 and BODIPY-BAK/Bcl-x_L. Tris buffer was used for FITC labelled BID peptides in direct binding experiments with Mcl-1 and Bcl-x_L. Comparison of titrations between buffers indicate similar values. Assays were run in 96 or 384 well Optiplates and were scanned using a Perkin Elmer EnVision™ 2103 MultiLabel plate reader. Fluorescein labelled peptides used an excitation and emission wavelength of 480 (30) nm and 535 (40) nm respectively (dichroic mirror 505 nm) whilst BODIPY labelled peptides used an excitation and emission wavelength of 531 (25) nm and 595 (60) nm respectively (dichroic mirror 555 nm),

BODIPY-BAK/Bcl-x_L competition assays

BODIPY-BAK/Bcl-x_L competition assays were performed in 384 well plates in phosphate buffer with the concentration of the inhibitor typically starting from 5-50 µM, diluted over 24 points in a 2/3 regime with [BODIPY-BAK] and [Bcl-x_L] fixed at 43 nM and 131 nM respectively. Plates were read after 1 h of incubation.

FITC-NOXA-B/Mcl-1 competition assays

FITC-NOXA-B/Mcl-1 competition assays were performed in either 96 well plates or 384 well plates in phosphate buffer, with the concentration of the inhibitor typically starting from 5-100 µM, diluted

over 24 points in a 2/3 regime and with [FITC-NOXA-B] and [Mcl-1] fixed at 50 nM and 150 nM respectively. Plates were read after 1 h.

FITC-BID/Mcl-1 and FITC-BID/Bcl-x_L direct binding assays

For all variants of the labelled BID peptides (FITC-BID-WT,FITC-BID-MM, FITC-BID-DM and FITC-BID-Aib), the direct binding assays were performed in Tris Buffer in 384 well plates. In protein titrations, the protein concentration started from between 1-10 µM and was diluted over 24 points in a 3/4 dilution regime, with the concentration of the labelled peptide fixed at 25-100 nM. In the peptide titrations, the concentration of the labelled peptides started from 5 µM and were diluted in a 2/3 regime over 24 points, with the concentration of the protein fixed at 50 nM. Plates were read after 2 hours of incubation, and read again after 4 and 18 hours to ensure complete equilibration.

Processing of fluorescence anisotropy data

Fluorescence anisotropy data was processed using Microsoft Excel to calculate intensity, anisotropy and fraction bound:

$$I = 2PG + S$$

$$r = \frac{S - PG}{I}$$

$$L_b = \frac{(r - r_{min})}{(\lambda(r_{max} - r) + r - r_{min})}$$

r = anisotropy, I = total intensity, P = perpendicular intensity, S = parallel intensity, Lb = fraction ligand bound, λ = I_{bound}/Iunbound , G = 1= instrumental factor.

This data was then transferred into Origin 8 which used to fit the data to either a logisitic model (for the calculation of IC_{50} and EC_{50}) or to the K_d model for the extraction of K_d values:

$$y = r_{min} + \frac{r_{max} - r_{min}}{1 + 10^{(x - logx_o)}}$$

Where for the logisitic model; y = r = anisotropy, x_o = mid-point of the curve between the r_{max} and r_{min} plateaux.

$$y = \frac{\{(K + x + [FL]) - \sqrt{\{(K + x + [FL])^2 - 4x[FL]\}}\}}{2}$$

For the K_d model; y = L_b*[FL], K = K_d, [FL] = Concentration of fluorescent ligand.

As noted in an earlier paper by us,[12] fluorescence anisotropy competition assays including those used here tend not to follow a simple 1:1 competition arising as a consequence of non-specific interactions of the tracer (fluorescently labelled peptide) permitting determination of only an IC_{50}.

This is caused by the additional hydrophobicity of the fluorescein group resulting in a non-unity hill coefficient; the extent to which this occurs differs for different competitor peptide sequences.

Supplementary Fluorescence Anisotropy assays

Table S1. *Fluorescence anisotropy competition and direct binding experiments for unconstrained BH3 peptides demonstrating agreement with previous published data*

	**Protein**	
**BH3 Peptide**	**Bcl-x$_L$ K_d**	**Mcl-1 K_d**
BODIPY-BAK	4 ± 3.7 nM	no binding
FITC-BID	21 ± 6.6 nM	50 ± 20 nM
FITC-NOXA-B	no binding	18.7 ± 0.9 nM
	**Bcl-x$_L$/ BODIPY-BAK IC$_{50}$**	**Mcl-1/ FITC-NOXA-B IC$_{50}$**
BAK	> 50 μM	no inhibition
BID	1.44 ± 0.05 μM	0.39 ± 0.08μM
NOXA-B	no inhibition	0.70 μM
BAD	0.14 ± 0.015 μM	no inhibition
BIM	0.32 ± 0.07 μM	0.05 ± 0.002 μM

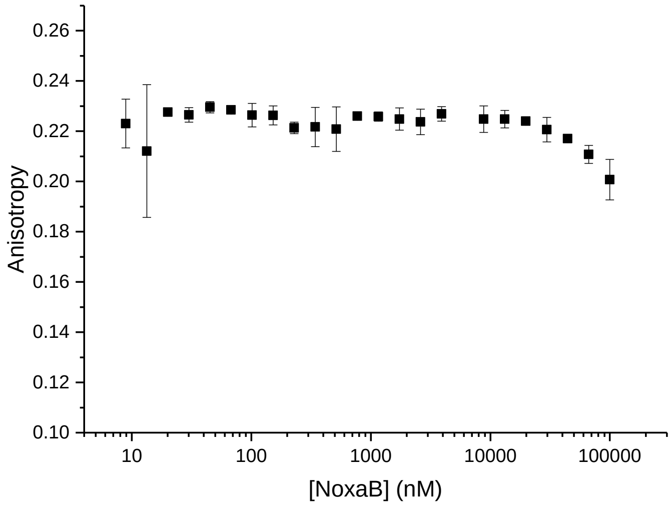

Figure S2. Fluorescence anisotropy competition assay for the inhibition of the BAK/Bcl-x$_L$ interaction by NOXA-B.

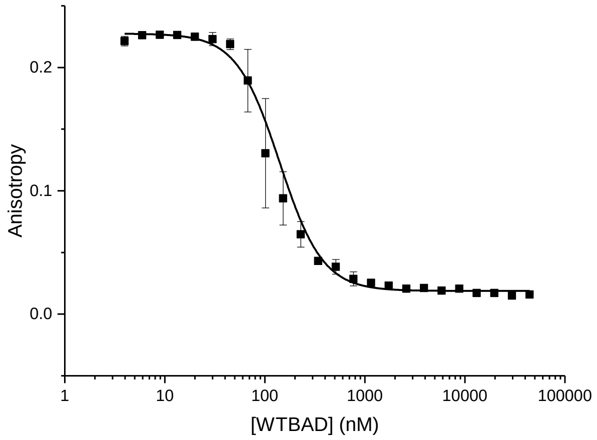

Figure S3. Fluorescence anisotropy competition assay for the inhibition of the BAK/Bcl-x_L interaction by BAD.

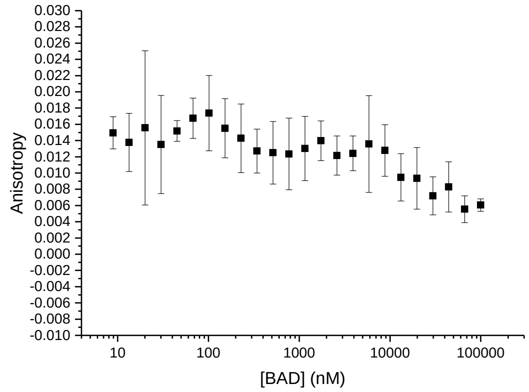

.

Figure S4. Fluorescence anisotropy competition assay for the inhibition of the Mcl-1/FITC-NOXA-B interaction by BAD.

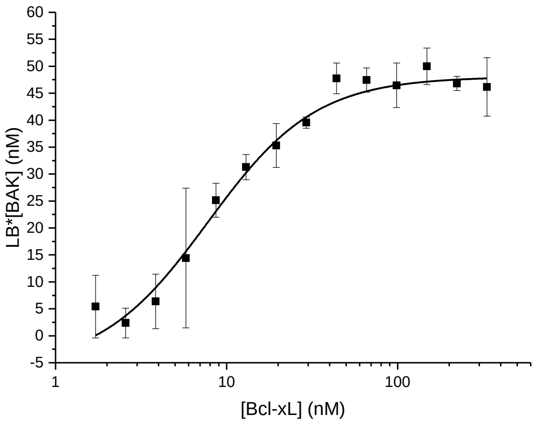

Figure S5. Fluorescence anisotropy titration assays for the addition of Bcl-x_L to a constant concentration of BODIPY-BAK.

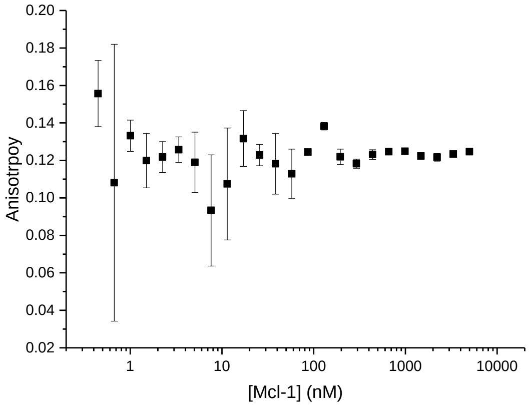

Figure S6. Fluorescence anisotropy titration assays for the addition of Mcl-1 to a constant concentration of BODIPY-BAK.

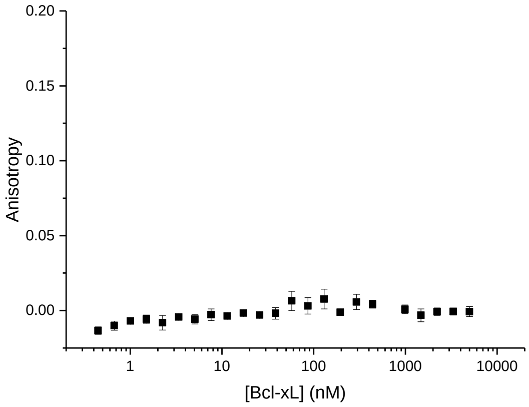

Figure S7. Fluorescence anisotropy titration assays for the addition of Bcl-x_L to a constant concentration of FITC-NOXA-B.

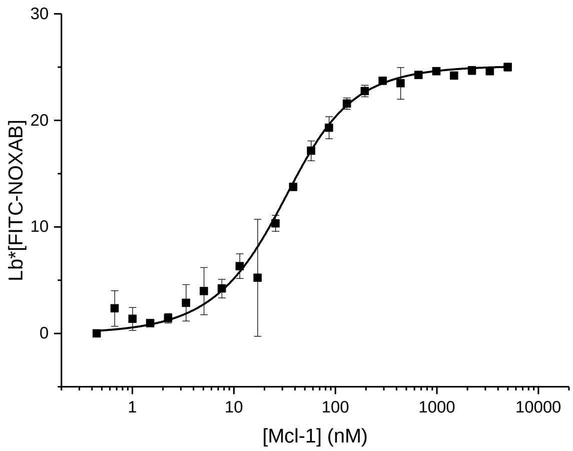

Figure S8. Fluorescence anisotropy titration assays for the addition of Mcl-1 to a constant concentration of FITC-NOXA-B.

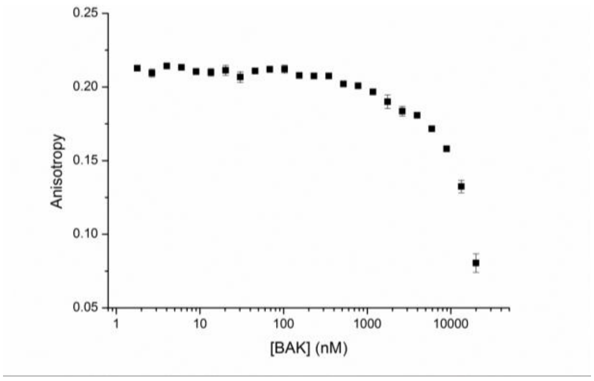

Figure S8. Fluorescence anisotropy competition assay for the inhibition of the BODIPY-BAK/Bcl-x_L interaction by BAK.

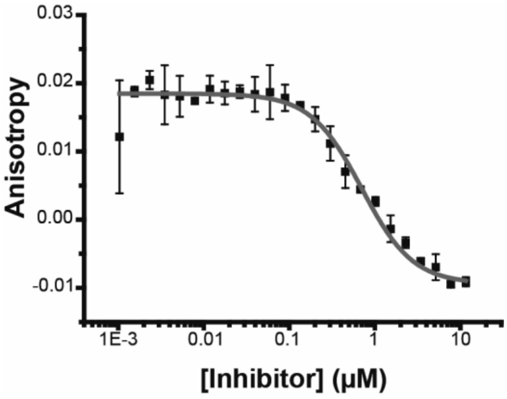

Figure S9. Fluorescence anisotropy competition assay for the inhibition of the Mcl-1/FITC-NOXA-B interaction by NOXA-B.

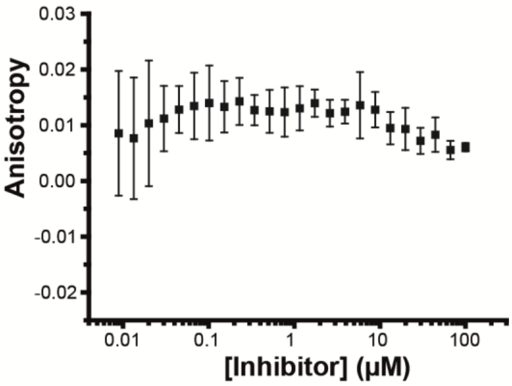

Figure S10. Fluorescence anisotropy competition assay for the inhibition of the Mcl-1/FITC-NOXA-B interaction by BAK.

Co-crystallisation

Mcl-1 (172-327) was incubated at a 1:1.1 ratio with BID-MM overnight at 4°C in 100mM TRIS pH 8.0, 200mM NaCl, 0.5mM DTT, before concentration of 12 mg/ml. Conditions were screened using the sitting-drop vapor-diffusion method with commercially available screens. Crystals grew in a 1:1 drop of 18% PEG 20,000, 0.1 M TRIS pH 8.0 at 4°C.

Bcl-x_L was incubated with an excess of BIM-MM overnight at 4°C before co-purification on in size exclusion chromatography in 50mM TRIS pH 8.0, 200mM NaCl, 0.5mM DTT. Conditions were screened using the sitting-drop vapor-diffusion method with commercially available screens. Crystals grew in a 1:1 drop of 12% Peg 1500, 0.1M Sodium Acetate pH 5.5, 2.5M NaCl, 1.5% MPD at 20°C from complex at 5mg/ml.

Hits were cryoprotected with glycerol (10% for Mcl-1/BID-MM, 20% for Bcl-x_L/BIM-MM), frozen in liquid N_2 and taken to Diamond Light Source for data collection. Data were collected at 100 K at 0.15° oscillations (Mcl-1/BID-MM) or 0.25° oscillations (Bcl-xl/BIM-MM). Data were processed with the xia2 bundle[13] running XDS[14] XSCALE, Pointless and Aimless.[15, 16] The last 770 images of the Bcl-xl/BIM-MM dataset were omitted from data processing due to radiation damage to the crystal. Phasing of the data by molecular replacement was achieved in Phaser[17] using chain A from 2NL9 as a search model for the Mcl-1/BID-MM structure, and chain A from 3FDL as a search model for Bcl-x_L/BIM-MM. Inspection of electron density maps was done in COOT[18] Initial refinement was with done in REFMAC[19], with further refinement using PHENIX[20] and analysis with Molprobity.[21] Restraints for the non-natural linkages in the ligands were generate in Lidia.[18] Table S1 shows statistics for data collection and refinement. (Mcl-1/BID-MM PDB ID: 5C3F and Bcl-x_L/BIM-MM PDB ID: 5C3G)

X-ray Data Collection and Refinement Statistics

**Protein**	Mcl-1 (172-327)	Bcl-x_L (1-198, missing 27-82)
**Ligand**	BID-MM	BIM-MM
Reservoir conditions	18% PEG 20,000, 0.1 M TRIS, pH 8.0	12% Peg 1500, 0.1M Sodium Acetate pH 5.5, 2.5M NaCl, 1.5% MPD
X-ray source	Diamond Light Source Beamline i03	DLS i02
**Data collection**		
Space group	P 21 21 2	P 63 2 2
**Unit cell dimensions**		
a, b, c (Å)	80.83, 37.02, 56.92	106.22, 106.22, 92.76
	90.00, 90.00, 90.00	90.00, 90.00, 120.00
Resolution (Å)	1.43 – 46.54 (1.467- 1.43)	2.45 – 34.76 (2.69 – 2.45)
Observations	134442	100497
Unique reflections	30488	11578
R merge	0.062 (0.682)	0.044 (0.872)
$\langle I \rangle / I\ \sigma(I)$	13.5	29.2 (3.39)
Completeness	99.00%	98.10%
Redundancy	4.2	8.7
**Refinement**		
Protein molecules in the a.u.	1	1
R_{work} / R_{free} (%)	16.7 / 19.6	22.6 / 27.0
**No atoms**		
Protein	2504	2188
Ligand	355	277
Water	190	3
**Mean B-factors (Å)**		
Protein	22.86	84.99
Ligand	32.11	88.78
Water	33.85	61.16
**R.m.s. deviations**		
Bond lengths (Å)	0.004	0.003
Bond angles	0.68	0.691
**Ramachandran statistics**		
% favoured	99.42	95.97
% allowed	0.58	4.03
% outliers	0	0
**PDB code**	5C3F	5C3G

Electron density of stapled regions

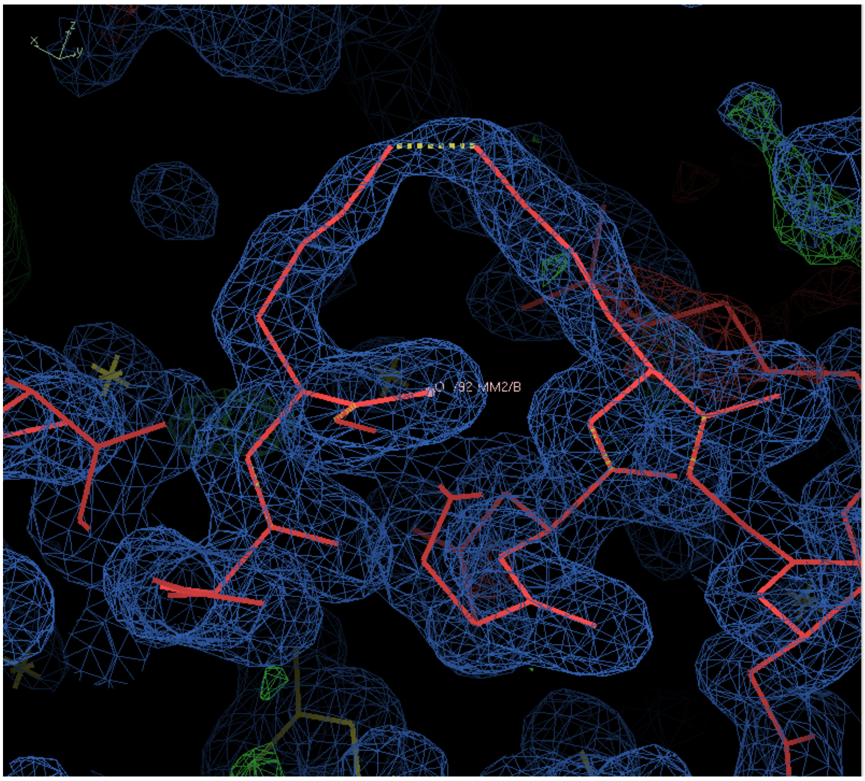

Figure S11: Electron density of region hydrocarbon staple in BID-MM bound to Mcl-1

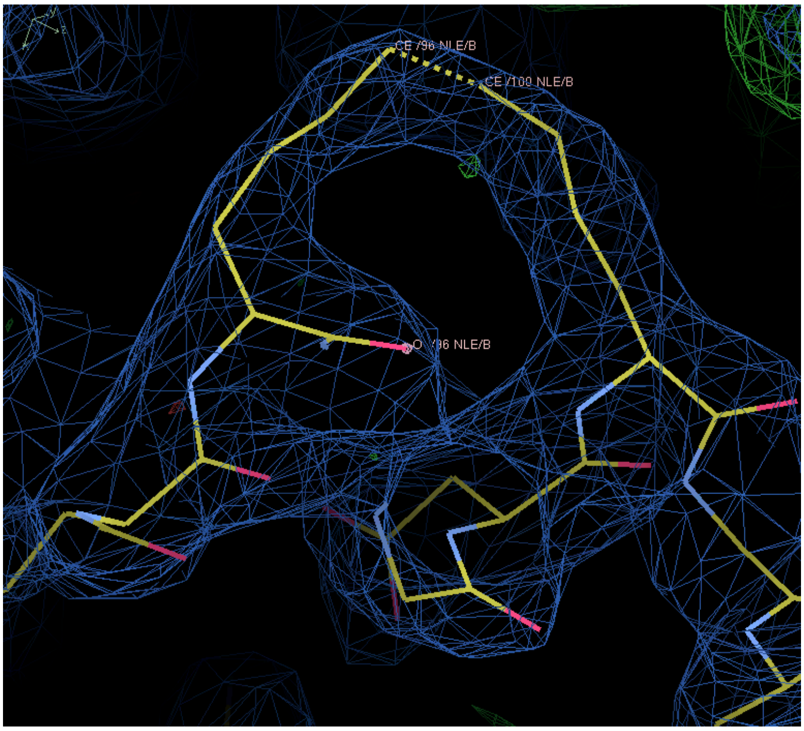

Figure S12: Electron density of region around staple in BIM-MM bound to Bcl-x_L

SPR of BIM series of peptides

The binding kinetics and affinities were analysed by SPR (Biacore 3000). His-SUMO Bcl-xl was immobilised on an Ni-NTA chip non-covalently in PBS with 0.01mg/ML BSA and 0.1% Tween 20 (Running buffer). The immobilization level of Bcl-x_L reached 1200 response units (RU). For the binding affinity assays, the peptides were prepared as 5mM stocks in 100% DMSO. Prior to analysis the peptides were diluted into running buffer. Experiments were run at 40ul/min in running buffer. The peptides were injected over the Bcl-x_L and reference surfaces for 180 seconds, followed by a 20 minute dissociation period. The binding kinetic parameters were evaluated with BIA-Evaluation software (BIAcore 3000) in which all data sets were fit to a simple 1:1 (Langmuir) binding model.

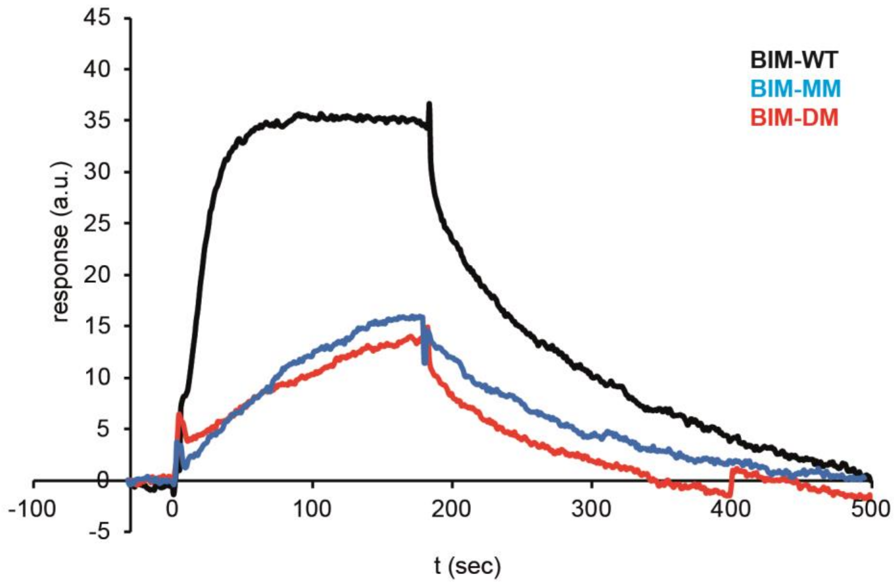

Figure S13: Sensorgram of 100nM BIM peptides flowed over immobilised Bcl-x_L.

Van’t Hoff analysis of direct binding van't Hoff analysis of fluorescence anisotropy data

The van't Hoff analysis for the fluorescently labelled BID peptides was performed in 50 mM HEPES pH 7.5, 200mM NaCl, 0.05% Tween 20, because the pH of TRIS changes with temperature; this is reasonable as the Kd / EC_{50}’s are similar between HEPES and TRIS. The protein concentration started from between 2.5-10 µM and was diluted over 24 points in a 2/3 dilution regime, with the concentration of the labelled peptide (FITC-BID-WT, FITC-BID-MM) fixed at 25nM and 50nM respectively. After incubation of the plate until the lower anisotropy plateau had equilibrated (~20 hours), the plates were scanned at 18 °C, then the temperature in the plate reader increased in steps of 5 °C, with a 10 minute equilibration time before the plate was read at each increasing temperature. The data was processed as described previously and plotted as ln K_a against 1/T (K).

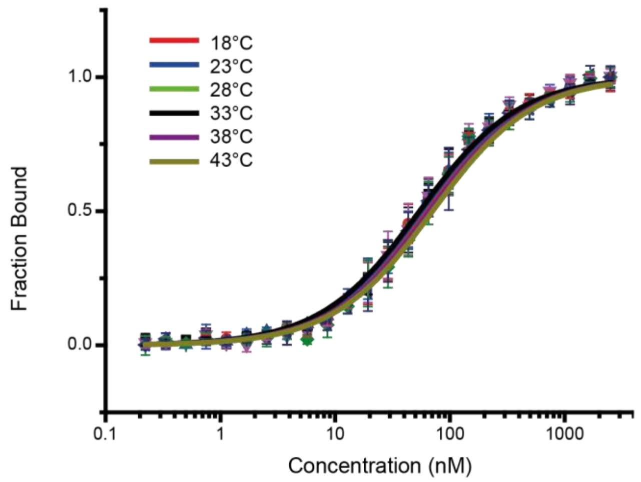

Figure S14: Fluorescence anisotropy titration assays for the addition of Bcl-x_L to a constant concentration of FITC-BID, recorded at increasing temperatures and fitted to a Kd model. Performed in 50 mM HEPES pH 7.5, 200 mM NaCl, 0.05% Tween 20.

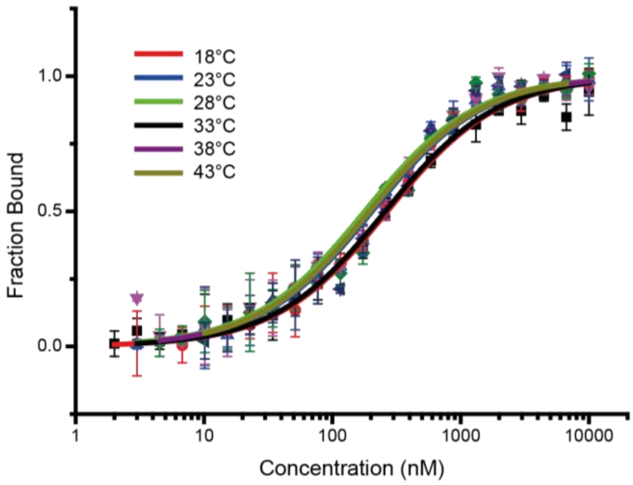

Figure S15: Fluoresence anisotropy titration assays for the addition of Bcl-x_L to a constant concentration of FITC-BID-MM, recorded at increasing temperatures and fitted to a Kd model. Performed in 50 mM HEPES pH 7.5, 200mM NaCl, 0.05% Tween 20.

Sequences of protein constructs

His-SUMO-Bcl-x_L (1-198, missing residues 27-82) Sumo tag removed with Ulp1 during purification.

```
atgggcagcagccatcatcatcatcatcacagcagcggcctggtgccgcgcggcagccat
 M  G  S  S  H  H  H  H  H  H  S  S  G  L  V  P  R  G  S  H
atgtcggactcagaagtcaatcaagaagctaagccagaggtcaagccagaagtcaagcct
 M  S  D  S  E  V  N  Q  E  A  K  P  E  V  K  P  E  V  K  P
gagactcacatcaatttaaaggtgtccgatggatcttcagagatcttcttcaagatcaaa
 E  T  H  I  N  L  K  V  S  D  G  S  S  E  I  F  F  K  I  K
aagaccactcctttaagaaggctgatggaagcgttcgctaaaagacagggtaaggaaatg
 K  T  T  P  L  R  R  L  M  E  A  F  A  K  R  Q  G  K  E  M
gactccttaagattcttgtacgacggtattagaattcaagctgatcagacccctgaagat
 D  S  L  R  F  L  Y  D  G  I  R  I  Q  A  D  Q  T  P  E  D
ttggacatggaggataacgatattattgaggctcacagagaacagattggtggaatgtct
 L  D  M  E  D  N  D  I  I  E  A  H  R  E  Q  I  G  G  M  S
cagtctaaccgtgaactggttgttgacttcctgtcttacaaactgtctcagaaaggttac
 Q  S  N  R  E  L  V  V  D  F  L  S  Y  K  L  S  Q  K  G  Y
tcttggtctcagatggctgctgttaaacaggctctgcgtgaagctggtgacgaatttgaa
 S  W  S  Q  M  A  A  V  K  Q  A  L  R  E  A  G  D  E  F  E
ctgcgttaccgtcgtgctttctctgacctgacctctcagctgcacatcaccccgggtacc
 L  R  Y  R  R  A  F  S  D  L  T  S  Q  L  H  I  T  P  G  T
gcttaccagtctttcgaacaggttgttaacgaactgttccgtgacggtgttaactggggt
 A  Y  Q  S  F  E  Q  V  V  N  E  L  F  R  D  G  V  N  W  G
cgtatcgttgctttcttctctttcggtggtgctctgtgcgttgaatctgttgacaaagaa
 R  I  V  A  F  F  S  F  G  G  A  L  C  V  E  S  V  D  K  E
atgcaggttctggtttctcgtatcgctgcttggatggctacctacctgaacgaccacctg
 M  Q  V  L  V  S  R  I  A  A  W  M  A  T  Y  L  N  D  H  L
gaaccgtggattcaggaaaacggtggttgggacaccttcgttgaactgtacggtaacaac
 E  P  W  I  Q  E  N  G  G  W  D  T  F  V  E  L  Y  G  N  N
gctgctgctgaatctcgtaaaggtcaggaacgttaa
 A  A  A  E  S  R  K  G  Q  E  R  -
```

His-SUMO-Mcl-1 (172-327) Sumo tag removed with Ulp1 during purification.

```
atgggcagcagccatcatcatcatcatcacagcagcggcctggtgccgcgcggcagccat
 M  G  S  S  H  H  H  H  H  H  S  S  G  L  V  P  R  G  S  H
atgtcggactcagaagtcaatcaagaagctaagccagaggtcaagccagaagtcaagcct
 M  S  D  S  E  V  N  Q  E  A  K  P  E  V  K  P  E  V  K  P
gagactcacatcaatttaaaggtgtccgatggatcttcagagatcttcttcaagatcaaa
 E  T  H  I  N  L  K  V  S  D  G  S  S  E  I  F  F  K  I  K
aagaccactcctttaagaaggctgatggaagcgttcgctaaaagacagggtaaggaaatg
 K  T  T  P  L  R  R  L  M  E  A  F  A  K  R  Q  G  K  E  M
gactccttaagattcttgtacgacggtattagaattcaagctgatcagacccctgaagat
 D  S  L  R  F  L  Y  D  G  I  R  I  Q  A  D  Q  T  P  E  D
ttggacatggaggataacgatattattgaggctcacagagaacagattggtggatccgag
 L  D  M  E  D  N  D  I  I  E  A  H  R  E  Q  I  G  G  S  E
ttgtaccggcagtcgctggagattatctctcggtaccttcgggagcaggccaccggcgcc
 L  Y  R  Q  S  L  E  I  I  S  R  Y  L  R  E  Q  A  T  G  A
aaggacacaaagccaatgggcaggtctggggccaccagcaggaaggcgctggagacctta
 K  D  T  K  P  M  G  R  S  G  A  T  S  R  K  A  L  E  T  L
cgacgggttggggatggcgtgcagcgcaaccacgagacggccttccaaggcatgcttcgg
 R  R  V  G  D  G  V  Q  R  N  H  E  T  A  F  Q  G  M  L  R
aaactggacatcaaaaacgaagacgatgtgaaatcgttgtctcgagtgatgatccatgtt
 K  L  D  I  K  N  E  D  D  V  K  S  L  S  R  V  M  I  H  V
ttcagcgacggcgtaacaaactggggcaggattgtgactctcatttctttggtgccttt
 F  S  D  G  V  T  N  W  G  R  I  V  T  L  I  S  F  G  A  F
gtggctaaacacttgaagaccataaaccaagaaagctgcatcgaaccattagcagaaagt
 V  A  K  H  L  K  T  I  N  Q  E  S  C  I  E  P  L  A  E  S
atcacagacgttctcgtaaggacaaaacgggactggctagttaaacaaagaggctgggat
 I  T  D  V  L  V  R  T  K  R  D  W  L  V  K  Q  R  G  W  D
gggtttgtggagttcttccatgtagaggacctagaaggtggctaa
 G  F  V  E  F  F  H  V  E  D  L  E  G  G  -
```

References

1. D. J. Yeo, S. L. Warriner and A. J. Wilson, *Chem. Commun.*, 2013, **49**, 9131-9133.
2. G. Sheldrick, *Acta Crystallographica Section A*, 2008, **64**, 112-122.
3. H. Flack, *Acta Crystallographica Section A*, 1983, **39**, 876-881.
4. E. Kaiser, R. L. Colescott, C. D. Bossinger and P. I. Cook, *Anal. Biochem.*, 1970, **34**, 595-598.
5. J. Marik, A. Song and K. S. Lam, *Tet. Lett.*, 2003, **44**, 4319-4320.
6. M. Certo, V. D. G. Moore, M. Nishino, G. Wei, S. Korsmeyer, S. A. Armstrong and A. Letai, *Cancer cell*, 2006, **9**, 351-365.
7. A. Barnard, K. Long, D. J. Yeo, J. A. Miles, V. Azzarito, G. M. Burslem, P. Prabhakaran, T. A. Edwards and A. J. Wilson, *Org. Biomol. Chem.*, 2014, **12**, 6794-6799.
8. J. Fremaux, L. Mauran, K. Pulka-Ziach, B. Kauffmann, B. Odaert and G. Guichard, *Angew. Chem. Int. Ed.*, 2015, **54**, 9816-9820.
9. P. Luo and R. L. Baldwin, *Biochemistry*, 1997, **36**, 8413-8421.
10. A. Barnard, K. Long, D. J. Yeo, J. A. Miles, V. Azzarito, G. M. Burslem, P. Prabhakaran, A. E. T and A. J. Wilson, *Organic & biomolecular chemistry*, 2014, **12**, 6794-6799.
11. D. J. Yeo, S. L. Warriner and A. J. Wilson, *Chemical communications*, 2013, **49**, 9131-9133.
12. J. P. Plante, T. Burnley, B. Malkova, M. E. Webb, S. L. Warriner, T. A. Edwards and A. J. Wilson, *Chem. Commun.*, 2009, 5091-5093.
13. G. Winter, *J. Appl. Crystallogr.*, 2010, **43**, 186-190.
14. W. Kabsch, *Acta Crystallographica Section D*, 2010, **66**, 125-132.
15. P. Evans, *Acta Crystallographica Section D*, 2006, **62**, 72-82.
16. P. R. Evans, *Acta Crystallographica Section D*, 2011, **67**, 282-292.
17. A. J. McCoy, R. W. Grosse-Kunstleve, P. D. Adams, M. D. Winn, L. C. Storoni and R. J. Read, *J. Appl. Crystallogr.*, 2007, **40**, 658-674.
18. P. Emsley, B. Lohkamp, W. G. Scott and K. Cowtan, *Acta Crystallographica Section D*, 2010, **66**, 486-501.
19. A. A. Vagin, R. A. Steiner, A. A. Lebedev, L. Potterton, S. McNicholas, F. Long and G. N. Murshudov, *Acta Crystallographica Section D*, 2004, **60**, 2184-2195.
20. P. V. Afonine, R. W. Grosse-Kunstleve, N. Echols, J. J. Headd, N. W. Moriarty, M. Mustyakimov, T. C. Terwilliger, A. Urzhumtsev, P. H. Zwart and P. D. Adams, *Acta Crystallographica Section D*, 2012, **68**, 352-367.
21. V. B. Chen, W. B. Arendall, III, J. J. Headd, D. A. Keedy, R. M. Immormino, G. J. Kapral, L. W. Murray, J. S. Richardson and D. C. Richardson, *Acta Crystallographica Section D*, 2010, **66**, 12-21.
